# Review of Polycyclic Aromatic Hydrocarbons (PAHs) Sediment Quality Guidelines for the Protection of Benthic Life

**DOI:** 10.1002/ieam.4142

**Published:** 2019-06-22

**Authors:** Joy A McGrath, Namita Joshua, Amanda S Bess, Thomas F Parkerton

**Affiliations:** ^1^ Exponent, New York New York USA; ^2^ HDR, Mahwah New Jersey USA; ^3^ Chevron Energy Technology Company Houston Texas USA; ^4^ ExxonMobil Biomedical Sciences Spring Texas USA

**Keywords:** Sediment quality guidelines (SQGs), Polycyclic aromatic hydrocarbons (PAHs), Equilibrium partitioning, Passive sampling, Toxic units

## Abstract

Polycyclic aromatic hydrocarbons (PAHs) in sediments can pose harm to the benthic community. Numerous sediment quality guidelines (SQGs) for the protection of benthic life are available to assess the risk of individual PAHs and PAH mixtures in sediments. Sediment quality guidelines are derived using empirical or mechanistic approaches. Empirically based guidelines are derived using databases of paired sediment chemistry and biological responses and relating sediment concentration to the frequency of an adverse response. Mechanistically based SQGs are derived by considering the inherent aqueous toxicity of the chemical to different biota coupled with site‐specific sediment characteristics (i.e., organic C) known to influence PAH bioavailability. Additionally, SQGs are derived to be either protective or predictive of adverse effects in benthic organisms. The objective of this critical review was to evaluate SQGs for use in screening‐level risk assessments to identify sediments that may pose a risk to the benthic community. SQGs for PAHs were compiled and compared, and performance evaluated for predicting the presence and absence of toxicity using an extensive field data set. Furthermore, a 2‐carbon equilibrium partitioning model and direct measurement of porewater via passive sampling were evaluated for improved performance in higher tiered risk assessments. Recommendations for the use of SQGs in screening evaluations, enhancements to current approaches, and opportunities to refine site risk estimate assessments using passive sampling measurements are discussed. *Integr Environ Assess Manag* 2019;15:505–518. © 2019 SETAC

## Key Points

- Mechanistic guidelines intended to be protective of effects of PAH mixtures to benthos are preferred for risk screening.
- Guidelines intended to be predictive of effects exhibited variable performance.
- Risk evaluation can be practically refined using improved partitioning model or passive sampling measurements.

## INTRODUCTION

Polycyclic aromatic hydrocarbons (PAHs) are ubiquitous contaminants in the environment exhibiting moderate to low water solubility that promotes sorption to particulates and subsequent accumulation in sediments. Sources of PAHs to the aquatic environment include releases during transportation or industrial use of petroleum, wastewater effluent discharge, and combined sewer overflows and urban runoff as well as natural seeps. In addition, PAHs are generated from coal and oil combustion, which can contribute to sediment contamination, particularly in the vicinity of former manufactured gas plant (MGP) sites. Polycyclic aromatic hydrocarbon mixtures resulting from oil (petrogenic) or combustion (pyrogenic) sources have different characteristic PAH profiles with petrogenic sources containing a higher percentage of alkylated compounds (Neff 1979).

Sediment quality guidelines (SQGs) for protection of benthic life are compound‐ (e.g., naphthalene) or class‐specific (total PAHs) estimates of concentrations in sediments that are intended to protect sediment‐dwelling organisms from adverse effects. Two approaches have been used to derive SQGs: empirically and mechanistically based approaches (alternatively, association‐ and causality‐based SQGs, respectively). Empirically based SQGs are evaluated from concurrent measurements of chemical concentrations in lab‐or field‐collected sediments and different types of biological effects data. These data are used to determine chemical concentrations that are either protective or predictive of adverse effects on benthic organisms, as inferred from the absence or presence of effects found in association with chemical concentrations measured in corresponding sediments. Such association‐based SQGs rely on the implicit assumption that any chemical detected in a sediment observed to have an adverse effect on benthic organisms is causing the observed effect, even if this is not the case. An advantage of empirically derived SQGs is that derivation often makes use of readily available bulk (i.e., dry basis) sediment concentration data. However, a clear disadvantage is that application over a wide range of sediment types, without consideration of site‐specific sediment characteristics, will likely lead to an SQG value that is unnecessarily conservative for some sediments (see USEPA 2000 and 2005 for examples). Another disadvantage of empirically based guidelines is that known differences in toxicity of individual PAHs or PAH mixtures are ignored. Mechanistically based SQGs are derived by considering the inherent aqueous toxicity of the chemical to a range of aquatic biota coupled with the site‐specific sediment characteristics known to influence bioavailability). For PAHs, equilibrium partitioning–based (EqP‐based) SQGs consider the fraction of organic C (*f*_OC_) in sediment to be a key factor dictating partitioning between the bulk sediment, porewater, and tissues of benthic organisms. Because benthic organisms have been shown to exhibit a similar range of sensitivity to that of water column organisms (Di Toro et al. 1991; Redman et al. 2014), a water quality criterion (WQC) developed to be protective of aquatic life that is applied to sediment porewater should provide a level of protection to benthic organisms comparable to that of water column organisms.

For a particular chemical, the equilibrium relationship between the OC‐normalized sediment concentration (C_SOC_ ~ µg/g_OC_) and the freely dissolved porewater concentration (C_*W*_ ~ µg/L) are related to the sediment OC partition coefficient (*K*_OC_), defined as follows:

(1) $$K_{\text{OC}}=\frac{C_{\text{SOC}}}{C_{W}}.$$

When a WQC is substituted for the porewater concentration, the resulting sediment concentration is equivalent to a sediment quality criterion (SQC). Note that for purposes of the present paper, SQG and SQC are interchangeable.

(2) $${\text{SQC}}_{\text{OC}}=K_{\text{OC}}\text{WQC}.$$

The objectives of the present review are to identify and compare SQGs for PAHs for the protection of benthic life that have been derived using different methods and data sets. Recommendations for the use of SQGs in screening evaluations, enhancements to current approaches, and opportunities to refine site risk assessments using a 2‐carbon (2‐C) EqP model or passive sampling measurements to establish sediment remediation goals are discussed.

## METHODOLOGY AND APPROACH

Sediment quality guidelines for individual and total PAH were compiled. Each guideline was categorized by derivation method (i.e., empirical or mechanistic) and objective (i.e., protective or predictive). Protective guidelines are concentrations below which adverse effects are unlikely. In contrast, predictive guidelines are concentrations intended to define hazard thresholds above which adverse effects are likely. The data and methods used for guideline derivation were reviewed. The present review included the types of biological effect data (e.g., species, endpoints, duration), chemical data, statistical methodology, and geographic region the data represent. This information was summarized and guidelines were grouped by objective and compared (Supplemental Data Table S1).

The performance of empirical SQGs were evaluated for use in screening‐level risk assessments to identify sediments in which contaminants are unlikely to pose a risk to the benthic community. Additionally, mechanistic SQGs were also evaluated to determine whether PAHs could be the causative agent of adverse effects (USEPA 2012). Because multiple PAHs occur in sediments, SQGs that consider PAH mixtures, either through a total PAH value or through an additive toxic unit approach, were compared. This evaluation involved the analysis of an independent field data set that was not included in the derivation of any of the SQGs. The data set was compiled from 19 different former MGP and smelter facilities that included both PAH sediment chemistry and toxicity data from 187 sediment samples (Arp et al. 2015). In these sediments, PAHs are likely to serve as the main contributor to the toxicity, given the source of the contamination. The data set included total organic carbon (TOC), black carbon (BC), and concentrations of 34 individual PAHs in the bulk sediment and porewater (as determined via passive sampling methods). Toxicity was measured in 28‐d mortality tests using *Hyalella azteca.* Each SQG for PAH mixtures was tested to determine the percentage of correct predictions as well as false positives and negatives using the matrix for assessing SQG performance provided in Table 1.

**Table 1:** Criteria for assessing sediment quality guideline performance

Sample concentration	Not toxic	Toxic
Below guideline	Correct result	False negative
Above guideline	False positive	Correct result

A false positive occurs when toxicity is expected but not observed. It should be noted that some empirical SQGs were developed to identify sediments that were not toxic (i.e., protective SQGs) and concentrations above these guidelines does not necessarily mean toxicity is expected, but rather toxicity becomes more likely as the magnitude above these thresholds increases. As a simplification for the present analysis, nontoxic samples that exceeded these protective SQGs are labeled as “false positive” even though exceedance of these SQGs does not imply that toxicity is expected. Additional testing is recommended to confirm absence or presence of effects. In contrast, a false negative occurs when toxicity is not expected but is observed. Recent approaches to improve the predictive ability of EqP‐based PAH benchmarks by further improving bioavailability estimates were evaluated, including use of a revised model that incorporates BC as a second partitioning phase as well as direct measurement of freely dissolved porewater concentrations using passive sampling methods.

## RESULTS

### Empirically based SQGs

Sediment quality guidelines differ in the data used for derivation, the statistical approach taken, and the objective. A summary of guidelines reviewed for PAHs is provided in the Supplemental Data and Table S1. Eight protective guidelines were identified using empirically based methods (TEL, T20, TEC, ERL, LEL, SCO, ISQG, and UET), and 3 protective guidelines were reported using mechanistically based methods (ESBs, MPC, and SRC). Eight predictive guidelines were derived using empirically based methods including CSL, SEL, SLC, ERM, AET, MEC, PEC, and PEL. The SRC was the only predictive, mechanistically based guideline.

### Dry weight–normalized SQGs for individual PAHs

Several approaches have been proposed to develop empirically based SQGs. One of the earliest examples is the screening‐level concentration (SLC) approach, which is based on the observed association of the presence or absence of benthic organisms with chemical concentrations measured in marine sediments (Neff et al. 1986, 1987). Briefly, a probability distribution of sediment chemical concentrations at sites where a particular organism occurs is first created. The 90^th^ percentile of this distribution (for *N* ≥ 20) is defined as the species screening‐level concentration (SSLC). The species should be able to survive at sediment concentrations less than the SSLC. The SSLCs for the various organisms for which sufficient data were available were then used to construct an SSLC probability distribution (1 data value that characterizes hazard per species). Analogous to the approach used to derive WQC (Stephan et al. 1985), the 5^th^ percentile concentration of this distribution defined the SLC. Sediment chemical concentrations below the SLC should be protective of most (i.e., 95%) benthic species. The perceived strength of the approach was the use of available field data that did not require an a priori assumption about the distribution of benthic organisms and chemical concentrations in field sediments.

Long and Morgan (1990, 1991) and Long et al. (1995) used a similar approach to characterize the probability of effects associated with different threshold concentrations. Using only effects data, they established an effects range low (ERL), corresponding to the 10^th^ percentile of effects data, as a concentration below which effects are infrequently observed. Similarly, at concentrations exceeding the effects range median (ERM), corresponding to the 50^th^ percentile of effects concentrations, effects are frequently observed (i.e., more often than not).

The approach of Long and Morgan (1990, 1991) was extended to include quantal data on absence and presence of effects (MacDonald 1992). It was envisioned that this might better define the risk associated with elevated levels of chemical stressors in sediments. MacDonald (1992) defined both a threshold effects level (TEL), the geometric mean of the 15^th^ percentile of the effects data and the 50^th^ percentile of the no‐effects data, and a probable effects level (PEL), the geometric mean of the 50^th^ percentile of effects data and 85^th^ percentile of no‐effects data. Although there were differences in these early derivation methods, the various approaches were found to yield similar thresholds (Long and MacDonald 1992). The ERLs and TELs and ERMs and PELs tended to agree to within a factor of 2. These guidelines have been subsequently updated as additional data became available and extended for use in different contexts and regions. For example, the original ERLs and ERMs have been extended to additional chemicals in both freshwater and marine sediments (MacDonald 1992; Ingersoll et al. 1996). Similarly, the TEL and PEL approach has been used to derive SQGs for both marine and freshwater sediments (e.g., Ingersoll et al. 1996; MacDonald et al. 1996; Smith et al. 1996). Menchaca et al. (2014) applied the ERL/ERM and TEL/PEL approaches to derive sediment protection levels for the Basque Coast of Spain.

Another example of empirical SQGs are apparent effects thresholds (AETs) developed for the Puget Sound area using paired chemistry and toxicity data from field survey data. The AET was identified as the highest chemical concentration in the sediment samples that did not demonstrate any significant effects relative to the controls. Apparent effects thresholds were originally developed for 17 individual PAHs for specific species and endpoints (e.g., amphipods, oyster larvae, bacteria) in 1988 (Barrick et al. 1988) and then reevaluated in 1994 (Gries and Waldow 1996). The State of Washington Department of Ecology (WDE) used the AET approach to establish SQGs by taking the lowest AET for each PAH and species, which were either the AETs for echinoderm larvae or Microtox^®^ (bacteria). The WDE developed revised guidelines (see sediment cleanup objective [SCO] and cleanup screening level [CSL] in Table S1 and Supplemental Data) using regional data following the AET protocols and expressed the guidelines on an OC basis (Washington State Department of Ecology 2015). The revised guidelines are used to help identify sites that need to be remediated and/or to establish sediment cleanup levels.

### Dry weight–normalized SQGs for PAH mixtures

Several empirical methods have been used to derive SQGs for total PAHs based on sum concentration of parent PAHs (see Table 2). The protective SQGs ranged from 197 to 4022 µg/kg. The predictive SQGs were more variable ranging from 1 500 to 100 000 µg/kg. A likely reason for the orders of magnitude variability is the differing role that parent PAHs contribute to observed effects coupled with uncertain bioavailability of PAHs across sites. This large concentration range highlights a major disadvantage of using an empirical, dry weight‐normalized approach, because it assumes the total parent PAH is responsible for the observed effects and ignores not only the different toxicity and bioavailability of individual parent PAHs but also the potential contributing role of alkyl PAHs as well as other stressors.

**Table 2:** Sediment guideline values for empirically based methods: Protective (µg/kg)

PAH	ERL	LEL	TEL	TEL	TEL	TEL	T20	TEC	TEC	SCO^a^
Source	Long et al. 1995	Persaud et al. 1993	MacDonald et al. 1996	Ingersoll et al. 1996	CCME 1999	Menchaca et al. 2014	Field et al. 2002	MacDonald et al. 2000	Swartz 1999	WDE 2015
Acenaphthene	16	—	6.71	—	6.71	—	19	—	—	16
Acenaphthylene	44	—	5.87	—	5.87	—	14	—	—	66
Anthracene	85.3	220	46.9	10	46.9	—	34	57.2	—	220
Benzo[*a*]anthracene	261	320	74.8	15.72	31.7	14.2	61	108	—	110
Benzo[*a*]pyrene	430	370	88.8	32.4	31.9	9.8	69	150	—	99
Chrysene	384	340	108	27	57.1	8.9	82	166	—	110
Dibenzo[*a*,*h*]anthracene	63.4	60	6.22	10	6.22	3.9	19	33	—	12
Fluoranthene	600	750	113	31.46	111	14.4	119	423	—	160
Fluorene	19	190	21.2	10	21.1	—	19	77.4	—	23
Naphthalene	160	—	34.6	14.65	34.6	5.5	30	176	—	99
Phenanthrene	240	560	86.7	18.73	41.9	13.3	68	204	—	100
Pyrene	665	490	153	44.27	53	18.9	125	195	—	1000
Benzo[*b*]fluoranthene	—	240	—	—	—	5.8	130	—	—	—
Benzo[*k*]fluoranthene	—	240	—	27.2	—	—	70	—	—	—
Benzo[*g*,*h*,*i*]perylene	—	170	—	15.5	—	5.1	67	—	—	31
Indeno[1,2,3‐*cd*]pyrene	—	200	—	17	—	6.5	68	—	—	34
Dibenzofuran	—	—	—	—	—	—	—	—	—	15
Total PAHs^b^	4022	4000	1684	264.1	—	197	—	1610	2900	—

CCME = Canadian Council of Ministers of the Environment; ERL = effects range low; LEL = lowest effect level; OC = organic carbon; SCO = sediment cleanup objective; SQG = sediment quality guideline; TEC = threshold effects concentration; TEL = threshold effects level; T20 = chemical concentration corresponding to 20% probability of observing toxicity; WDE = Washington State (US) Department of Ecology.

^a^ Presented on a mg/kg OC basis as in WDE 2015.

^b^ Toxicity data that went into deriving total PAH SQGs is variable. For example, ERL derivation includes data sets where a minimum of 4 PAHs and a maximum of 21 PAHs were quantified. The majority of the data sets include 13 to 16 parent PAHs.

Swartz (1999) developed a consensus‐based approach SQG for total PAHs that combined empirical and mechanistic guidelines (see Supplemental Data for more details). Swartz (1999) expressed the chemical concentrations on an OC‐normalized basis in an attempt to better account for bioavailability. The mean threshold effect concentration (TEC) for total PAH below which effects are not expected was 290 µg/g_OC_. The mean extreme effects concentration above which effects are likely was 10 000 µg/g_OC_. Using PAH sediment chemistry and empirical lab toxicity data for 10‐d exposure to *Ampelisca abdita* obtained in a monitoring program, the lower threshold effect concentration for total PAH performed poorly in being protective of effects. Toxicity was observed below a total PAH concentration of 290 µg/g_OC_ in many samples and was attributed to the presumed confounding influence of other stressors present in the field‐collected samples (Swartz 1999). No samples had a total PAH concentration greater than 10 000 µg/g_OC._

### Mechanistically based SQGs

Mechanistically based SQG approaches are based on either extrapolating substance‐specific aqueous toxicity test data using the EqP framework or sediment toxicity data. The EqP approach was applied initially by the United States Environmental Protection Agency (USEPA) to 3 individual PAHs for deriving sediment guidelines based on respective WQC. Swartz and coworkers provided a significant advancement when they directly accounted for the additivity of toxic effects caused by 13 individual parent PAHs that compose a PAH mixture (Swartz et al. 1995). They computed the toxic unit (TU) for each PAH in the PAH mixture by dividing the predicted porewater concentration calculated using EqP by the corresponding effect concentration in water (i.e., lethal concentration to 50% test organisms, LC50). The total TUs of the mixture were then computed by summing up the TUs of the individual PAHs.

The USEPA followed a similar approach as Swartz et al. (1995) to derive the equilibrium partitioning sediment benchmarks (ESBs) for PAH mixtures (USEPA 2003) by including the theory of additivity via TUs to account for the effects of the mixture. The ESBs were derived using the Target Lipid Model (TLM) for 34 PAHs, which included not only parent PAHs but also alkylated homologs, which are not included in empirical SQGs for total PAHs. The TLM is a mechanistic approach that can be used to derive aquatic and sediment protection values for hydrocarbons (Di Toro et al. 2000; Di Toro and McGrath 2000; Redman et al. 2014). An important factor in the TLM approach is that it makes use of a species sensitivity distribution (SSD) and an acute‐to‐chronic ratio (ACR) in a way that is analogous to USEPA methods used to derive WQC. This ensures that a TLM‐based SQG will be protective of most species and that the level of protection will apply to both acute and chronic effects.

Environmental risk limits originally proposed in 2001 (Verbruggen et al. 2001) have been recently updated by the Netherlands National Institute for Public Health and the Environment (RIVM) (Verbruggen 2012). Maximum permissible concentrations (MPCs) for 16 individual PAHs (in water, sediment, and soil) were derived and used to set Dutch environmental quality standards. The MPCs are intended to provide concentrations below which no negative effect on ecosystems is expected with values derived using a recognized framework in the Netherlands (EC 2003; Van Vlaardingen and Verbruggen 2007). Sediment MPCs were derived from available sediment toxicity data with application of assessment factors dependent on the type and amount of data available (e.g., acute versus chronic, species representation). If toxicity data were limited or unavailable, then a sediment value was computed using EqP and a water‐derived MPC. The lower of the 2 values was selected as the sediment MPC. For marine environments, an additional precautionary assessment factor of 10 was applied to the freshwater sediment MPC. The sediment MPCs were derived on an OC basis and then presented on a milligrams per kilogram dry weight basis, assuming 10% organic matter (5.88% OC), which is the Dutch standard. Serious risk concentrations (SRC) were also derived from ecotoxicity data and assessment factors to represent concentrations at which an intervention may be required.

The advantages of the MPC approach are that it makes use of water and sediment toxicity data, it considers bioavailability, and it uses a standardized method for computing environmental risk limits intended to be protective of benthic life. The disadvantages are the use of arbitrary assessment factors and the consideration of toxicity endpoints that are confounded by other variables (e.g., ultraviolet light), which may not be relevant for SQGs and thus introduce considerable unwarranted conservatism. In addition, the MPC values were developed for only 16 parent PAHs, and the framework itself did not address toxicity of PAH mixtures.

Sediment quality criteria for 16 PAHs based on a classification system for protection of sediments in Norway have been proposed but not adopted (Bakke et al. 2010). In that framework, concentrations of contaminants below their respective chronic predicted no‐effect concentration (PNEC) were considered to have no adverse impacts on the benthic community. The sediment chronic PNEC was derived using methods similar to those used by Verbruggen (2012) such that sediment toxicity data were used if available or established from a water PNEC and EqP relationship. The methods for computing a PNEC followed Technical Guidance Document (TGD) protocols, which include application of assessment factors depending on toxicity data availability (EC 2003). The sediment PNECs were derived on an OC basis and then presented on a milligrams per kilogram dry weight basis, assuming 1% OC, which is considered typical for Norwegian sediments.

### Comparison of empirical and mechanistic PAH SQGs

The empirical SQGs for PAHs intended to be protective and predictive are provided in Tables 2 and 3, respectively. Mechanistic guidelines for PAHs are summarized in Table 4. Empirical guidelines are expressed on a dry weight basis (e.g., micrograms chemical per kilogram dry weight) except where noted. Mechanistic guidelines are expressed on an OC basis (e.g., milligrams per kilogram OC).

**Table 3:** Sediment guideline values for empirically based methods: Predictive (µg/kg)

PAH	ERM	SEL	AET	PEL	PEL	PEL	T50	PEC	CSL^a^
Source	Long et al. 1995	Persaud et al. 1993	Barrick et al. 1988	Menchaca et al. 2014	MacDonald et al. 1996	Ingersoll et al. 1996	Field et al. 2002	MacDonald et al. 2000	WDE 2015
Acenaphthene	500	—	130	4.5	89	—	116	—	57
Acenaphthylene	640	—	71	—	128	—	140	—	66
Anthracene	1100	3700	280	39.6	245	167	290	845	1200
Benzo[*a*]anthracene	1600	14 800	960	135	693	285	466	1050	270
Benzo[*a*]pyrene	1600	14 400	1100	125	763	320	520	1450	210
Chrysene	2800	4600	950	114	846	406	650	1290	460
Dibenzo[*a*,*h*]anthracene	260	1300	230	43.9	135	28.2	113	—	33
Fluoranthene	5100	10 200	1300	193	1494	319	1034	2200	1200
Fluorene	540	1600	120	17.4	144	150	114	540	79
Naphthalene	2100	—	230	31.7	391	140	217	561	170
Phenanthrene	1500	9500	660	97.3	544	410	455	1170	480
Pyrene	2600	8500	2400	181	1398	493	932	1520	1400
Benzo[*b*]fluoranthene	—	13 400	1800	168	—	—	1107	—	—
Benzo[*k*]fluoranthene	—	13 400	1800	—	—	158	537	—	—
Benzo[*g*,*h*,*i*]perylene	—	3200	670	79.9	—	252	497	—	78
Indeno[1,2,3‐*cd*]pyrene	—	3200	600	101	—	240	488	—	88
Dibenzofuran	—	—	110	—	—	—	—	—	58
Total PAHs^b^	44 792	100 000	—	1500	16 770	3370	—	22 800	—

AET = apparent effects threshold; CSL = cleanup screening level; ERM = effects range medium; OC = organic carbon; PEC = probable effects concentration; PEL = probable effects level; SEL = severe effect level; SQG = sediment quality guideline; T50 = chemical concentration corresponding to 50% probability of observing toxicity; WDE = Washington State (US) Department of Ecology.

^a^ Presented on a mg/kg OC basis as in WDE 2015.

^b^ Toxicity data that went into deriving total PAH SQGs is variable. For example, ERM derivation includes data sets where a minimum of 4 PAH and a maximum of 21 PAH were quantified. The majority of the data sets include 13 to 16 parent PAHs.

**Table 4:** Sediment guideline values for mechanistically based methods (mg/kg OC)

PAH	MPC^a^ freshwater	ESB mixed	PNEC^b^ marine/estuarine
Protection level	Low	Low	Low
Source	Verbruggen 2012	USEPA 2003	Bakke et al. 2010
Acenaphthene	16	491	16
Acenaphthylene	2.9	452	3.3
Anthracene	0.80	594	3.1
Benzo[*a*]anthracene	6.0	841	6.0
Benzo[*a*]pyrene	8.3	964	42
Chrysene	27	843	28
Dibenzo[*a*,*h*]anthracene	0.31	1122	59
Fluoranthene	70	708	17
Fluorene	14	539	26
Naphthalene	29	385	29
Phenanthrene	13	597	50
Pyrene	29	698	28
Benzo[*b*]fluoranthene	13	979	24
Benzo[*k*]fluoranthene	13	980	21
Benzo[*g*,*h*,*i*]perylene	8.3	1095	2.1
Indeno[1,2,3‐*cd*]pyrene	6.5	1115	4.7
Total PAHs	—	—	200

ESB = equilibrium partitioning sediment benchmark; MPC = maximum permissible concentration; OC = organic carbon; PNEC = probable no‐effect concentration.

^a^ Published on a mg/kg Dutch sediment standard; converted to mg/kg OC using Dutch standard of 10% organic matter (5.88% OC).

^b^ Published on a mg/kg basis by assuming 1% OC; converted to mg/kg OC using 1%.

**Figure 1:**
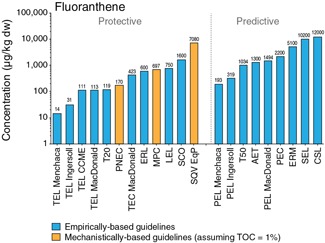
Comparison of guidelines for fluoranthene. Mechanistic guidelines were converted to dry weight basis assuming 1% OC. AET = apparent effects threshold; CCME = Canadian Council of Ministers of the Environment; CSL = cleanup screening level; ERL = effects range low; LEL = lowest effect level; MPC = maximum permissible concentration; OC = organic carbon; PEC = probable effects concentration; PEL = probable effects level; PNEC = probable no‐effect concentration; SEL = severe effect level; SCO = sediment cleanup objective; SQV = sediment quality value; TEC = threshold effects concentration; TEL = threshold effects level; T20 = chemical concentration corresponding to 20% probability of observing toxicity; T50 = chemical concentration corresponding to 50% probability of observing toxicity.

To compare empirical and mechanistic guidelines for the individual PAHs, the mechanistic guidelines were converted to a dry weight basis using 1% OC, which is a conservative assumption that results in the computation of a low dry weight concentration. Graphical displays comparing all guidelines on a micrograms per kilogram basis are shown in Supplemental Data Figures S1 to S17. A representative example is provided for fluoranthene in Figure 1. The guidelines are categorized based on the objective to be protective or predictive of benthic effects. Empirical and mechanistic fluoranthene SQGs intended to be protective range from 14 (TEL) to 1 600 (SCO) µg/kg and 170 (PNEC) to 7080 (ESB) µg/kg, respectively. For predictive guidelines, only empirically based values are available and range from 193 to 12 000 µg/kg.

Based on the comparison of the individual guidelines (Figure 1) (and related figures included as Supplemental Data), several observations are evident. Empirical SQGs are generally lower than the mechanistic guidelines. This is not unexpected, given that empirical SQGs for individual PAHs are often derived from field sediments that have many constituents present and the observed effect is most likely related to a combination of these constituents and/or other stressors and not to an individual PAH. In contrast, mechanistic guidelines derived from compound‐specific toxicity data (sediment or water) in which individual PAHs were the causative agent are expected to be higher because the derivation is not confounded by other stressors. An important observation is the 2 orders of magnitude in variation in empirically based SQG values. This variation highlights the dependence of this approach on the underlying field data set selected that provides the sediment chemistry and biological effects inputs used in SQG derivation. The TEL/PEL method yields the most conservative guidelines, which most likely results from considering sediment concentrations that had no observed effects. Similar general conclusions can be made for the other PAHs (Supplemental Data Figures S1–S17).

**Figure 2:**
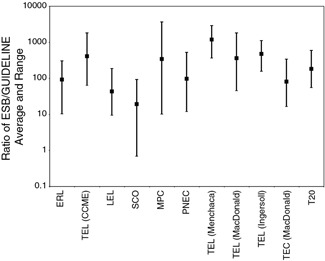
Ratio of ESBs to other guideline values. The datapoint is the average ratio of all PAHs for which a guideline was available and the range bars represent the minimum and maximum of the ratios for all PAHs which criteria were available. ERL = effects range low; ESB = equilibrium partitioning sediment benchmark; LEL = lowest effect level; MPC = maximum permissible concentration; PNEC = probable no‐effect concentration; SCO = sediment cleanup objective; TEL = threshold effects level; T20 = chemical concentration corresponding to 20% probability of observing toxicity.

For most PAHs, the USEPA's ESBs based on EqP and the TLM (USEPA 2003) represent the highest protective SQG values (assuming 1% OC). For each PAH, the ratio of the ESB to the other individual SQG values was calculated and the average, minimum, and maximum of all ratios are plotted in Figure 2. On average, ESBs are about 2 orders of magnitude higher than the other protective guidelines. The ESBs are higher than the other mechanistically based MPC and PNEC values because those values incorporate additional conservatism associated with default adjustment factors (ranging from 10 to 1 000) and other confounding variables such as ultraviolet light exposure, as previously explained. Furthermore, predictive empirically based SQGs are often below the ESB value (which is intended to be protective), highlighting the conservative nature of some empirically derived values.

### Use of SQGs for screening PAH mixture risks

The performance of empirical and mechanistic SQGs to correctly predict the absence or presence of toxicity in a tier I screening‐level risk assessment was evaluated using a data set for 187 sediment samples that had corresponding PAH chemistry and toxicity data to *H. azteca* (Arp et al. 2015). These sediment samples were collected from MGP sites where PAHs are expected to be the main causative agent for toxicity. Sediment quality guidelines selected for the present analysis focused on PAH mixtures rather than on individual PAHs (e.g., either total PAHs or TU approach). Empirical guidelines included those intended to be protective (ERL, lowest effect level [LEL], TEL, and TEC) or predictive (ERM, severe effect level [SEL], PEL, and PEC). Protective mechanistic guidelines tested were PNEC and ESB. Applying mechanistic SQGs will demonstrate whether the sediment‐associated PAHs are in fact contributing to the observed toxicity. Because the mechanistic SQGs are protective, an overestimation of toxicity can occur if the test organism is less sensitive than a 5^th^‐percentile species. In contrast, exceeding the empirical SQGs cannot be used to draw definitive conclusions regarding whether and to what extent PAHs in sediments are contributing to the observed benthic toxicity.

For each guideline, the correct predictions for absence or presence of toxicity and the percents false positive and false negative were computed by comparing the SQGs to the total PAH sediment concentration. The empirical SQGs for total PAH use a subset of indicator PAHs to predict the likelihood of toxicity from all PAH and other co‐occurring contaminants present in the sediments used to derive the guidelines. The subset of indicator PAHs are PAHs identified as priority pollutants and can include up to 16 PAHs (Keith and Telliard 1979); therefore, only those PAHs were included in the sum total PAH sediment concentration for evaluation of these empirical guidelines. The PNEC was also based on the 16 priority pollutant PAHs and as such only those were considered. In contrast, the ESB method was evaluated using 34 measured PAHs, which includes alkylated homologs of parent PAHs, and are expected to best capture different sources of PAH present in field‐collected samples. Performance metrics for each SQG used in risk screening are summarized in Table 5 with further details in Table S2.

**Table 5:** Reliability of sediment quality guideline

Guideline	% Correct predictions (both absence and presence of effects)	% False negative	% False positive
Performance of protection guidelines (tier 1 screening)			
ERL	35.3	1.1	63.6
LEL	35.3	1.1	63.6
TEL (Ingersoll et al. 1996)	25.1	0	74.9
TEC (MacDonald et al. 2000)	30.5	0.5	69.0
PNEC	27.3	6.4	66.3
ESB‐EqP	36.4	0.5	63.1
Performance of protection guidelines (tier 2 evaluation)			
ESB‐EqP+BC	85.0	12.8	2.1
Passive sampling	74.9	1.0	24.1
Performance of predictive guidelines (tier 1 screening)			
ERM	65.2	2.1	32.6
SEL	75.9	4.3	19.8
PEL (Ingersoll et al. 1996)	34.8	1.1	64.2
PEC (MacDonald et al. 2000)	55.1	1.6	43.3

BC = black carbon; ERL = effects range low; ERM = effects range medium; ESB‐EqP = equilibrium partitioning sediment benchmark; LEL =lowest effect level; PEC = probable effects concentration; PEL = probable effects level; PNEC = probable no‐effect concentration; TEC = threshold effect concentration; TEL = threshold effects level.

The protective SQGs performed similarly. All protective SQGs had a low percentage (<6.4%) of false negatives (i.e., concentration below the SQG but toxic), which supports use in initial risk screening. However, only 25.1% to 36.4% of samples were correctly classified as nontoxic or toxic using the protective SQGs. This result is not unexpected, given that protective guidelines were not developed to identify samples that are not likely to be toxic. Additionally, the protective SQGs exhibited a high percentage of false positives (i.e., concentration above SQG but nontoxic) ranging from 63.1% to 74.9% confirming that these guidelines are conservative and, when exceeded, may overstate risks to benthos. This result is also not unexpected, given that the protective guidelines were not intended to predict effects for a specific species, but to be protective of 95% of species.

Guidelines intended to be predictive of effects exhibited variable performance. Correct predictions (refer to Table 1) improved, compared to protective SQGs, and ranged from 34.8% to 75.9%. The percentage of samples that were below predictive SQGs but were toxic (i.e., false negatives) was low, ranging from 1.1% to 4.3%. The percentage of samples exceeding predictive SQGs and not exhibiting toxicity (i.e., false positives) ranged from 19.8% to 64%. The high percentage of false positives indicates that the threshold effect concentrations that are used to establish predictive SQGs are conservative when compared to toxicity endpoints investigated in the present study. Thus, even when predictive SQGs are exceeded, sediment toxicity may not be observed. This highlights the practical concern with using predictive guidelines to establish sediment cleanup goals.

The preceding analysis suggests that the empirical ERL and mechanistic ESB SQGs intended to be protective performed equally well at predicting the lack of toxicity in this data set and would be best suited for use in screening‐level assessments. However, because the ESB takes into account the toxicity and composition of both parent and alkylated PAHs as well as OC content of the sediment, which provides a preliminary consideration of sediment‐specific partitioning behavior, this approach is preferred given its mechanistic basis. Despite the potential utility of applying mechanistic ESB for initial risk screening of PAH‐contaminated sediments, it is important to highlight that for protective guidelines more than 63% of the sediments in the validation data set are incorrectly classified as toxic (false positives) (Table 5). One reason for this discrepancy is that, by design, the ESBs are protection values below which toxicity is expected to occur in only 5% of the most sensitive species; thus, an exceedance of the ESB is not meant to predict toxicity. Our findings are consistent with previous studies that have applied the ESB method to assess risks from toxicity from PAHs in sediments and found that many of the sediments that exceeded the ESB were not toxic (Ozretich et al. 2000; West et al. 2001; Kreitinger et al. 2007; Kane Driscoll et al. 2009; McDonough et al. 2010; Tuikka et al. 2016).

**Figure 3:**
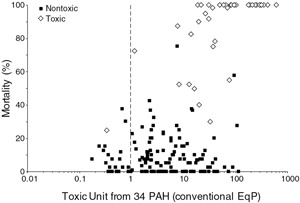
Mortality of *Hyalella azteca* (28‐d) as a function of sediment toxic units computed from 34 PAHs. The toxic units were based on conventional equilibrium partitioning that considers natural OC as the sole C‐binding phase. OC = organic carbon.

The ESBs were evaluated for predicting toxicity of sediments using a data set that has 187 data points with corresponding PAH chemistry and toxicity to *H. azteca* (Arp et al. 2015). The methodology of McDonough et al. 2010 was followed*.* Figure 3 compares the observed percent survival of *H. azteca* as a function of total sediment TUs computed using the ESBs for 34 PAHs. With the exception of 1 sample, toxic sediments with 50% or less survival had total TUs > 1 and the majority had total TUs ≥ 8; thus, the ESBs correctly predicted toxicity in all but 1 toxic sample. The high TUs are partly the result of using the ESB, a protection value below which effects to 5% of species are expected. The test species *H. azteca* is about a factor of 3 less sensitive than the 5^th^‐percentile value upon which the ESBs are based (McGrath and Di Toro 2009). Although the ESBs performed well at correctly identifying toxic sediments, the method performed poorly at identifying nontoxic sediments. Many of the sediments that were not toxic had total TUs ranging from 1 to 100 indicating toxicity was overestimated.

### Enhancements to higher tier risk evaluations

The EqP–ESB approach is an established framework for evaluating sediments for potential toxicity that considers bioavailability of nonionic organic contaminants such as PAHs (Kane Driscoll and Burgess 2007). However, as demonstrated in the previous section, this approach can be overly protective such that sediments that exceed the ESB SQG are not necessarily toxic and may not exhibit toxicity when tested using standardized sediment toxicity tests. As discussed in USEPA (2003), other types of C may be present at some site sediments in varying amounts (e.g., soot, char, coal ash), collectively referred to as “black carbon” (Goldberg 1985). Black carbon is a term that collectively consists of soot, coke, and charcoal‐like material and was found to contribute 15% to 30% of TOC in marine sediments (Middelburg et al. 1999) and on average 9% in 300 aquatic sediments (Koelmans et al. 2006). In contrast, natural organic carbon (NOC) consists of plant and animal debris and humic matter and is typically measured as TOC. Black carbon has a stronger binding affinity for PAHs than does NOC (Gustafsson and Gschwend 1997; Jonker and Koelmans 2002; Ghosh et al. 2003; Cornelissen and Gustafsson 2004; Vinturella et al. 2004; Lohmann et al. 2005). The presence of BC further reduces the porewater concentration of the PAH due to the higher partitioning coefficient, which results in lower bioavailability and toxicity. Thus, when a partitioning coefficient (e.g., OC partitioning, *K*_OC_) is used to compute porewater concentration in the EqP framework based solely on NOC (see Equation (2)), the porewater concentration may be significantly overestimated. Thus, in the absence of a reliable partitioning model that accounts for BC or other highly sorptive phases (e.g., weathered oil), OC‐normalized PAH sediment concentrations may not accurately predict true bioavailability.

Accounting for alternative sorptive phases for partitioning in the EqP framework and equations should improve the ability to predict dissolved porewater concentrations and ultimately the associated toxicity. Alternatively, freely dissolved chemical concentrations of the 34 PAHs in porewater can be directly quantified, thereby avoiding uncertainties associated with partitioning between the heterogeneous OC phases in site sediments and porewater. These 2 alternative improvements, 1) consideration of BC as a second partitioning phase and 2) use of measured porewater concentrations in lieu of OC‐normalized sediment concentrations, offer opportunities to improve the predictive ability of the EqP method for assessing bioavailability and toxicity of PAH mixtures in sediments.

### Black C partitioning model

Considerable literature are available to support the incorporation of BC partitioning into the EqP model to improve predictability (Gustafsson et al. 1996; Accardi‐Dey and Gschwend 2002, 2003; Cornelissen and Gustafsson 2004; Vinturella et al. 2004; Lohmann et al. 2005; Koelmans et al. 2006). Using this framework, PAHs bind to both NOC and BC but by different mechanisms. The binding to NOC is an absorption mechanism that follows a linear relationship as described by conventional EqP. The binding to BC is an adsorption process that can be described by a Freundlich isotherm. The combined total partitioning takes the form

(3) $$K_{d}\;=\;f_{\text{OC}}K_{\text{OC}}\;+\;f_{\text{BC}}K_{\text{BC}}C_{W}\left(n\;-\;1\right)$$

where *K*_d_ = solid‐water distribution coefficient, L/kg solid; *f*_OC_ = weight fraction of natural OC (kg OC/kg solids); *K*_OC_ = OC partition coefficient (L/kg_OC_); *f*_BC_ = weight fraction of BC (kg BC/kg solids); *K*_BC_ = BC partition coefficient (L/kg_BC)_; C_*W*_ = freely dissolved water concentration (µg/L); *n* = Freundlich coefficient for each PAH.

The solid‐water  distribution coefficient from Equation (3) is substituted into Equation (2) to yield

(4) $$C_{S}\;=\;{\text{CW}(f}_{\text{OC}}K_{\text{OC}}\;+\;f_{\text{BC}}K_{\text{BC}}C_{W}(n-1)).$$

Accardi‐Dey and Gschwend (2002) applied Equation (4) to assess the sorption of both NOC and BC in Boston Harbor, Massachusetts, USA sediments. In subsequent work, the importance of BC partitioning of PAHs was investigated in Boston and New York harbors (Lohmann et al. 2005). The dual sorption model was found to be a better predictor of overall sediment–water partitioning than the single sorption model.

**Figure 4:**
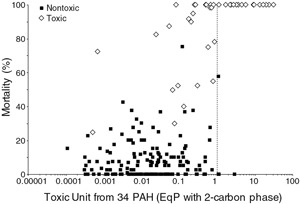
Mortality of *Hyalella azteca* (28‐d) as a function of toxic units predicted from the 2‐C model. Porewater toxic units computed from 34 PAHs measured by SPME. 2‐C = 2‐carbon; SPME = solid‐phase microextraction.

McDonough et al. (2010) also tested the 2‐C model for its ability to predict toxicity of PAHs in sediment samples in our validation data set. Values of *K*_BC_ for each PAH were based on a quantitative structure–activity relationship derived from a limited amount of data. A single *n* value of 0.7 was used for all PAHs. The porewater concentrations were predicted from sediment concentrations using Equation (4). The mortality of *H. azteca* as a function of total TUs based on the 2‐C model is shown in Figure 4. Fewer samples were predicted to be toxic with this model than the 1‐phase C EqP model, and most samples that were not toxic were correctly predicted to be not toxic (Table 5). However, some samples (12.8%) that were found to be toxic were predicted to be not toxic (i.e. were false negatives). Based on these results, adsorption of the PAH to BC appeared to be overestimated. However, the data were from 9 different sites, and using a single *K*_BC_ across sites is inconsistent with data from Hawthorne, Grabanski et al. (2007), which showed that measured *K*_BC_ values for individual PAHs vary by more than 2 orders of magnitude across different sites. These results are consistent with a field study by Brennan and Johnson (2017) who found improved estimates of PAH toxicity and bioaccumulation were observed with the 2‐C model compared to the 1‐carbon (1‐C) model, highlighting the importance of BC and the limitations of using a 1‐C phase model to describe PAH partitioning. Nevertheless, dissolved porewater predictions derived using the 2‐C model overpredicted the importance of BC partitioning in some sediments.

Although data suggest that partitioning of PAH is better described with a 2‐C model, applying this type of modeling framework in a risk assessment may require significant effort to reliably characterize site‐specific partitioning coefficients for *K*_BC_ and *K*_OC_ for multiple PAHs. Recent efforts have demonstrated that advances in partitioning modeling may be a feasible option (Davis and Di Toro 2015a, 2015b). However, direct porewater measurement may be a more practical solution for refined risk evaluations.

### Direct measurement of freely dissolved porewater concentrations

Sediment porewater concentrations (C_PW_) may be directly compared to water‐only benchmarks (effect concentrations or WQC, C_WQC_) as follows:

(5) $${\text{TU}}_{\text{pw}}\;=\;\frac{C_{\text{PW}}}{C_{\text{WQC}}},$$

where adverse effects to benthos are not expected if the magnitude of the porewater TU (or sum TU for PAH mixtures) is less than 1.0. WQC are derived from water‐only toxicity test results with low dissolved organic carbon (DOC) lab waters. As a result, most of the chemical is in a freely dissolved (not complexed by organic matter), bioavailable form. Two approaches are commonly utilized to obtain sediment porewater for analysis of hydrocarbon concentrations: 1) extraction of porewater from sediments or 2) passive sampling using either in‐situ or ex‐situ methods (Ghosh et al. 2014). Measurements of PAHs in extracted sediment porewater reflect the total porewater concentration, which is equal to the sum of freely dissolved and DOC‐complexed compound. The importance of DOC complexation increases for the less soluble, higher toxicity PAHs (Di Toro et al. 1991); thus, reliance on total measured porewater concentration can overstate bioavailability and predicted toxicity. A method of correcting for DOC complexation when calculating freely dissolved chemical in porewater measurements is available (USEPA 2012).

Passive sampling devices that quantify the freely dissolved concentrations are increasingly being applied to in‐situ and ex‐situ conditions to quantify the bioavailability of PAHs and other hydrophobic organics in sediments (Vinturella et al. 2004; Cornelissen et al. 2008; Lu et al. 2011; Burgess et al. 2015; Brennan and Johnson 2017). Passive sampling methods involve the partitioning of the chemical between the freely dissolved phase and an absorptive material. Methods have been developed that use various inert polymers such as polyethylene (PE), polyoxymethylene (POM), and polydimethylsiloxane (PDMS). A description of alternative passive sampling methods is beyond the scope of the present review. Descriptions of these methods, including discussions of the advantages and disadvantages of each, are available elsewhere (e.g., Hawthorne, Azzolina et al. 2007; Cornelissen et al. 2008; Gschwend et al. 2011; Mayer et al. 2014; Arp et al. 2015) and USEPA has included such methods in guidance for evaluating contaminated sediments (USEPA 2012, 2017a). An American Society for Testing and Materials (ASTM) method for determining porewater concentration of PAHs using solid‐phase microextraction (SPME) has also been developed (ASTM 2013, D7363‐13a). Furthermore, a comprehensive interlaboratory study of ex‐situ passive sampling of PAHs in sediments has been published, concluding that this measurement technique can be implemented to support risk assessment provided proper quality controls are included (Jonker et al. 2018).

**Figure 5:**
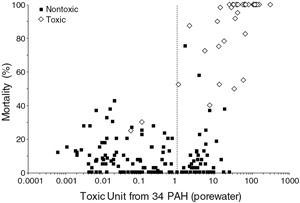
Mortality of *Hyalella azteca* (28‐d) as a function of porewater toxic units computed from 34 PAHs measured by SPME. SPME = solid‐phase microextraction.

McDonough et al. (2010) evaluated the ability of ex‐situ porewater measurements determined by passive sampling using SPME to predict the toxicity of the sediment samples. Porewater measurements for 34 PAHs were compared to the water‐only final chronic values for individual PAHs (USEPA 2003) using Equation (5) to calculate TUs. A comparison of *H. azteca* survival as a function of TUs based on porewater is shown in Figure 5. The ESBs based on porewater PAHs had the best overall performance of all of the SQGs (Table 5). With the exception of 2 samples, samples determined to be toxic had TUs greater than 1.0, which is a correct prediction. However, 24.1% of nontoxic samples also had a TU greater than 1.0, which represent false positives. The dose–response relationship based on porewater (Figure 5) is an improvement compared to using OC‐normalized sediment PAH concentrations and the ESB method based only on partitioning to NOC (Figure 3).

## DISCUSSION

Several SQGs for PAHs are available to evaluate risk to benthic organisms, and they fall into 2 categories: empirically or mechanistically derived. Depending on the threshold, they can be either protective or predictive of effects, which is important to recognize for proper interpretation in a risk assessment framework. Protective guidelines are often used in screening‐level risk assessments and represent concentrations below which minimal effects to the benthic community are expected. Predictive guidelines represent concentrations above which effects are more likely to occur. The EqP‐based ESB values yielded comparable or better performance than empirically based SQGs for screening risks in a large data set of sediments investigated in the present study. Furthermore, this approach has a number of advantages for use in screening evaluations, given 1) the underlying mechanistic basis of the derivation; 2) the ability to consider site‐specific profile and toxicity of both parent and alkylated PAHs; and 3) the attempt to take into account bioavailability through OC normalization.

Given the conservative nature of EqP–ESB, risk may be overstated, particularly when partitioning is underestimated by simple EqP assumptions based on OC normalization of PAH sediment concentrations. Modifications to the 1‐C phase conventional partitioning model to account for the presence of BC show promise in refining actual risk estimates. However, additional site data to characterize BC and PAH sorption characteristics is required for actual calibration and application of a 2‐C phase partitioning model.

The use of passive sampling methods provides the most direct way to quantify the bioavailability of PAH‐contaminated sediments and to refine risk estimates obtained via tier 1 screening evaluations. A broad consensus has been reached that porewater concentrations of PAHs as measured through passive techniques are better correlated with effects on benthos than bulk sediment concentrations are correlated with benthos effects (Mayer et al. 2014). Furthermore, the development of guidance for the use of passive sampling methods and standard protocols is leading to their growing use in site risk evaluations and acceptance by regulating agencies (USEPA 2017b). Consistent with the conclusions of Brennan and Johnson (2017), the use of direct porewater measurements determined through passive sampling techniques provides the logical path forward for refining benthic risk assessments, establishing improved remediation goals to protect benthic life, and assessing remedial effectiveness.

## Data Accessibility

Data and any associated metadata are available on request from the corresponding author Joy McGrath at jmcgrath@exponent.com. The data calculations are described to a level that can be replicated.

## SUPPLEMENTAL DATA

**Figure S1.** Comparison of guidelines for acenaphthene.

**Figure S2.** Comparison of guidelines for acenaphthylene.

**Figure S3.** Comparison of guidelines for dibenzo[*a*,*h*]anthracene.

**Figure S4.** Comparison of guidelines for benzo[*b*]fluoranthene.

**Figure S5.** Comparison of guidelines for benzo[*k*]fluoranthene.

**Figure S6.** Comparison of guidelines for anthracene.

**Figure S7.** Comparison of guidelines for benzo[*a*]anthracene.

**Figure S8.** Comparison of guidelines for fluorene.

**Figure S9.** Comparison of guidelines for naphthalene.

**Figure S10.** Comparison of guidelines for benzo[*g*,*h*,*i*]perylene.

**Figure S11.** Comparison of guidelines for indeno[1,2,3‐*cd*]pyrene.

**Figure S12.** Comparison of guidelines for benzo[*a*]pyrene.

**Figure S13.** Comparison of guidelines for chrysene.

**Figure S14.** Comparison of guidelines for phenanthrene.

**Figure S15.** Comparison of guidelines for pyrene.

**Figure S16.** Comparison of guidelines for dibenzofuran.

**Figure S17.** Comparison of guidelines for Total PAHs.

**Table S1.** Guidelines reviewed for PAHs.

**Table S2.** Protective guidelines accuracy test.

## Supporting information

This article includes online‐only Supplemental Data.

Supplementary information.
