# Supplementary material for: Review of Polycyclic Aromatic Hydrocarbons (PAHs) Sediment Quality Guidelines for the Protection of Benthic Life
Source: Integr Environ Assess Manag. 2019 Jun 22;15(4):505–18. doi: 10.1002/ieam.4142 (PMC6852300; doi:10.1002/ieam.4142)
Supplement: Supplementary file 1 — Supplementary information. [file IEAM-15-505-s001.docx]

# Supplemental Information

## **EMPIRICALLY BASED APPROACHES**

Apparent Effects Thresholds (AETs)

AET – chemical concentration of a contaminant above which statistically significant (p< 0.05) adverse effects of a particular biological indicator are always expected relative to appropriate reference controls. Concentrations are expressed as bulk chemical on a dry weight basis.

Apparent Effects Thresholds were developed for the Puget Sound (marine/estuarine) area using paired chemistry and toxicity data from survey data. The chemistry data were reviewed and had to meet certain quality standards for acceptance (e.g., blanks, standard reference materials, duplicates, matrix spikes). Bioassay data had to be performed with standard test protocols. Samples were identified as either impacted or not impacted when compared to controls. Samples were arranged with increasing chemical concentration. The not impacted sample with the highest concentration was identified as the AET. AETs were developed for 17 individual PAHs for specific species and endpoints (e.g., amphipods, oyster larvae, bacteria) in 1988 (Barrick et al. 1988) and then reevaluated in 1994 (Gries and Waldow 1996). The State of Washington has used these AETs to establish sediment quality guidelines by taking the lowest values for each PAH which were for either echinoderm larvae or Microtox^®^ (WDE 1990). However, WDE developed revised goals using data specific to the area (see SCO and CSL).

Advantages of this method are that it is easy to apply and makes no assumption of contaminant availability, which can also be considered a disadvantage. Some disadvantages of this method are that it relies on individual data points and outliers can heavily influence results, it cannot demonstrate a cause and effect relationship for any single chemical, and it can be under protective since it identifies the chemical concentration above which effects are always expected. The AETs provide moderate level of protection and should not be considered as sediment guideline values that protect 95% of species from adverse effects.

## ***Effects range – low and medium (ERL and ERM)***

ERL – concentration of chemical below which effects would be rarely observed

ERM – concentration of chemical at and above which effects would occur frequently

The ERL and ERM were developed using paired sediment chemistry and biological information from the U.S. National Status and Trends Program to develop an association between sediment chemistry and effects to benthic organisms. The data were reviewed for good chemical and biological reporting. The data were compiled from a variety of geographical locations (both marine and freshwater environments) in the United States and included species from different taxonomic groups at different life stages, different endpoints (mortality, measures of benthic community, histopathological disorders in fish) and data from field or spiked laboratory tests. In addition, the database included sediment guidance values derived from different methods (AET, EqP). The data were sorted in ascending order and the ERL and ERM were identified as the 10^th^ and 50^th^ percentiles, respectively. Values were provided for 13 individual PAHs. The ERL is a threshold concentration below which effects should rarely be observed, and as such, it can be considered appropriate for use as a sediment quality guideline that protects benthic organisms. The original values were derived by Long and Morgan (1991) and then revised in 1995 (Long et al. 1995). The same approach was applied to field collected data from freshwater sediments around the Great Lakes (Ingersoll et al. 1996) and marine/estuarine sediments around Florida (MacDonald 1992).

The advantages of this method are that it is easy to apply and makes no assumption of the contaminant availability, which can also be considered a disadvantage. In addition, the method can be considered a weight-of-evidence approach because it has been applied to sediments throughout the United States and considers a variety of species and endpoints. In this way, the values should be applicable across many sediments. The disadvantages are that the ERL and ERM are concentrations of the chemical that are associated with an effect, rather than causing the effect. In this way, the guidelines most likely are over protective because there were derived for sediments that had other contaminants present that could be contributing to the observed effects. In addition, the method does not make use of sediment samples that had no effects.

The ERL/ERM approach was applied to sediment samples collected from the Basque Coast in Spain using 756 sediment samples (Menchaca et al. 2014). Toxicity effects were mixed and included 48-hour embryo larval tests on urchin, 10-day mortality tests on amphipod, and Microtox^®^ and biotic index tests. ERL and ERM values were computed for 16 individual PAHs; however, in some cases sufficient data were not available to compute an ERL. The computed ERL values were greater than background levels and the methodology was deemed inappropriate for use in deriving sediment guidelines.

## ***Threshold effects level (TEL) and probable effects level (PEL)***

TEL – concentration of chemical below which effects would rarely be observed

PEL – concentration of a chemical equal to or above which effects would occur frequently

TEL and PEL were developed using the same chemistry and biological effects databases from the compilation of Long and Morgan for derivation of the ERL/ERM except the data were screened for Florida coastal waters (MacDonald 1992). The TEL and PEL are therefore for marine or estuarine sediments. The sediment concentrations associated with effects or no effects were separated and then ranked in ascending order. For a TEL and PEL to be computed, there must have been a minimum of 20 observations in both the effects and no effects data sets. The TEL was computed by taking the geometric mean of the 15^th^ percentile of the effects concentration data set and the 50^th^ percentile of the no effects concentration data set. The PEL was computed by taking the geometric mean of the 50^th^ percentile of the effects concentration data set and the 85^th^ percentile of the no effects concentration data set. The TEL was intended to represent a sediment protection value below which effects would rarely be observed. Values were provided for 13 PAHs.

The TEL/PEL approach was applied to other geographical regions. Smith et al. (1996) compiled data from freshwater sediments throughout North America including Canada. They compiled data from 56 publications where about 35% of the data came from the Great Lakes. The database included a variety of species (epibenthic and water column invertebrates) and endpoints (mortality, growth, community structure). Values were provided for six individual PAHs. In another application of the TEL/PEL method, values were computed for freshwater sediments for a specific endpoint of 28-day mortality to *H. azteca* (Ingersoll et al. 1996). CCME (1999) applied the TEL method to compute interim sediment quality guidelines for 13 individual PAHs. The National Status and Trends Program approach was followed such that paired sediment chemistry and effects data were compiled from sediments around North America. The toxicity data were from a variety of species and endpoints and went through a quality control screening.

The advantages of this method are that it is easy to apply and makes no assumption of the contaminant availability, which can also be considered a disadvantage. In addition, the method can be considered a weight-of-evidence approach because it has been applied to sediments throughout the United States and considers a variety of species and endpoints. In this way, the values should be applicable across many sediments. Another advantage of the statistical method is that it uses both effects and no effects data and by taking the geometric means, outliers cannot influence the results. The disadvantages are that the TEL and PEL are concentrations of the chemical associated with an effect, rather than causing the effect. In this way, the guidelines most likely are over protective because they were derived for sediments that had other contaminants present that could be contributing to the observed effects.

The TEL/PEL approach was applied to sediment samples collected from the Basque Coast in Spain using 756 sediment samples (Menchaca et al. 2014). Toxicity effects were mixed and included 48-hour embryo larval tests on urchin, 10-day mortality tests on amphipod, and Microtox^®^ and biotic index tests. TEL and PEL values were computed for 16 individual PAHs; however, in some cases sufficient data were unavailable to compute a TEL. The computed TEL values were greater than background levels and the methodology was deemed inappropriate for use in deriving sediment guidelines.

## ***Lowest effect level (LEL) and severe effect level (SEL)***

LEL – the sediment concentration of a chemical that can be tolerated by a majority of benthic species

SEL – the sediment concentration of a chemical that would be detrimental to the majority of benthic species

Sediment guidelines for the protection of benthic organisms were developed for sediments in Ontario based on the screening level concentration method proposed by Neff et al. (1987) that relates sediment chemistry and benthic community structure (Persaud et al. 1993). For each chemical and species, a frequency distribution of the sediment chemical concentration for all samples where the species was found is generated. The 90^th^ % of the distribution is identified as the SSLC, the species screening level concentration. The minimum requirement is 10 observations to compute an SSLC. A distribution of the SSLC is then generated and the 5^th^ and 95^th^ percentiles are identified as the LEL and SEL, respectively. The minimum data requirement to compute LEL and SEL is 20 species. In addition, data requirements included that the chemical concentrations should span two orders of magnitude, data should be representative of both clean and contaminated areas, and the species should be 75% benthic infaunal species rather than epibenthic species to ensure that the effects are from the sediment and not the water column. There were sufficient data to compute guidelines for 12 individual PAHs.

The advantages of this approach is that it takes into consideration all chronic responses because it is a cumulative measure of effects and does not rely on a specific endpoint. Another advantage is that by taking the 90^th^ %, extreme sediment concentrations are excluded. A disadvantage is that it does not determine a cause and effect relationship because it uses field data where a mixture of contaminants is present. Another disadvantage is that the range of chemical concentrations for a particular species can impact the results.

***Threshold effect concentration (TEC) and probable effect concentration (PEC) (individual PAHs)***

Existing sediment guidelines for the protection of freshwater sediments were reviewed for their commonalities. If they were derived from a paired sediment chemistry and effects database and their methods were transparent, they were considered in the compilation. Based on their narrative intent, the guidelines were grouped into two categories, concentrations below which harmful effects on sediment organisms were not expected (TEC) and concentrations above which harmful effects on sediment organisms occurred frequently (PEC) (MacDonald et al. 2000). A consensus-based guideline was computed by taking the geometric mean of the guideline values in each category. This was done for 10 individual PAHs. Using an independent dataset, the guidelines had 71–83% ability to correctly predict the absence of toxicity and greater than 92% ability to predict the presence of toxicity. The TEC was adopted by many state agencies (e.g., Florida, Minnesota, New Jersey, Oregon, Texas, Wisconsin, and Massachusetts) and federal agencies (e.g., National Oceanic and Atmospheric Administration [NOAA], USEPA Regions 3 and 5) as sediment guideline values to protect sediments against adverse impacts from exposure to contaminants.

The advantages include that any extreme values are dampened and it provides a weight-of-evidence approach since the various guidelines were developed using different databases, endpoints, geographical areas, and statistical method. The disadvantage is that there is no relationship between the chemical concentration and the effect.

## ***Midpoint effect concentration (MEC)***

The midpoint effect concentration is the midpoint concentration between the TEC and PEC (MacDonald et al. 2000). The MEC was computed to define levels of concern around the consensus-based guidelines for use in prioritizing sites for additional investigation (WDNR 2003). MECs were computed for 18 PAHs mostly based on consensus-based values from MacDonald et al. 2000. Other sources of sediment guidelines were used to fill in data gaps.

MECs should not be used as sediment guidelines. They offer no inherent level of protection.

## ***Upper effect threshold (UET)***

The UET is the lowest of the reliable AET values of all species. These values should not be used as sediment guideline values since they do not offer the same level of protection.

***Sediment cleanup objective (SCO) and cleanup screening level (CSL)***

SCO – no-adverse-effect level to the benthic community, used as a long-term goal

CSL – minor-adverse-effect level to the benthic community, used to identify sites that need clean up

The SCO and CSL were established by the State of Washington, Department of Ecology (WDE 2015). Paired chemistry and toxicity data from samples collected in marine sediments around the region were compiled. The toxicity data were for a variety of different tests, species, and endpoints. The criteria were developed using the AET approach (Barrick et al. 1988). SCO and CSL were derived for 17 individual PAHs. AETs were computed for each biological test and endpoint. The lowest AET was set to the SCO, and the second lowest was set to be the CSL. The advantages and disadvantages are similar to those for AETs.

## ***Logistic regression modeling T20 and T50 values***

T20 – chemical concentration corresponding to 20% probability of observing sediment toxicity

T50 – chemical concentration corresponding to 50% probability of observing sediment toxicity

The logistic regression modeling approach identifies the probability of observing adverse effects as a function of chemical concentration (Field et al. 2002). Paired chemistry and biological effects data from various databases (EMAP, NST, SEDQUAL) were compiled, including Atlantic, Pacific, and Gulf Coast data as well as marine and estuarine sediment data. Biological effects were 10-day amphipod toxicity data for mortality. Data sets were critically evaluated for collection and handling procedures, control responses, testing protocols, chemical measurements performed, etc. Data were screened such that if the concentration of a chemical in a toxic sample is similar to the concentration in a non-toxic sample, the sample was not included in the modeling approach. This was done because the chemical concentration in that sample could not have been causing the effect. The screened data were used to develop regression models relating chemical concentration to the probability of observing effects. The T20 and T50 values were identified for 22 individual PAHs. This methodology has not been adopted in a regulatory framework.

The advantages of this modeling approach are that it is based on a large database of chemistry and biological effects from coastal areas in North America and, as such, it is representative of many types of sediments and chemical contamination. In addition, the biological endpoints were consistent. Another advantage is that the user can identify any desired probability of effects based on established relationships. One disadvantage is that the modeling approach does not consider bioavailability of the contaminant. Another disadvantage is that the toxicity endpoints were 10-day mortality endpoints for amphipods rather than endpoints based on long-term exposure to chemical concentration or endpoints for lifecycle effects.

## ***Consensus-based threshold effect concentration and median effects concentration for total PAH***

Swartz (1999) made two observations regarding PAH sediment guidelines: 1) PAH sediment guidelines should be based on a mixture of PAHs and not individual PAHs, and 2) many marine sediment guidelines for PAHs have been proposed. Based on existing sediment guidelines, Swartz (1999) derived consensus-based SQCs for PAH mixtures for two categories of guidelines: threshold effect concentrations (TECs), which provide a high level of protection against adverse effects, and median effects concentrations, which provide a moderate level of protection against adverse effects. He combined empirical and mechanistic guidelines to demonstrate their similarity regardless of the method used to derive them. He derived PAH guidelines for total PAHs based on the sum of 13 PAHs. Empirical guidelines included ERL/ERM, TEL/PEL, SLC (Screening Level Concentration), and AET where individual PAH values were available. Next, sum total 13 PAHs were normalized to 1% organic carbon. The mechanistic guidelines included EqP guidelines for acenaphthene, phenanthrene and fluoranthene where an average was taken rather than a sum total based on 13 PAHs and guidelines derived from sigma PAH model. The mechanistic guidelines were expressed on an organic carbon basis. A consensus-based total PAH (based on 13 PAH) TEC was computed as the average of five guidelines (TEC, Equilibrium Partitioning (EqP), sigma PAH, ERL, and SLC). Similarly, a consensus-based total PAH MEC was computed as the average of four guidelines (PEC, AET, sigma PAH, and ERM). Consensus-based total PAH guidelines have units of µg/g_OC_ total PAH. When tested against field and laboratory data, the consensus-based TEC did well at predicting the lack of toxicity when PAHs were expected to be the main stressor in the sediment, and the MEC did well at predicting a moderate level of toxicity. However, when tested against sediment data collected in a monitoring program that contained samples from various sources, the TEC did not perform well at predicting the lack of toxicity. Toxicity was observed below the TEC, which was attributed to the presence of other stressors in the samples.

The advantages of the consensus based total PAH guidelines are that they consider the mixture of PAHs rather than single chemical approaches, are easy to apply, take bioavailability into account by normalization to organic carbon, and represent a weight-of-evidence approach since the various guidelines used in the derivation were based on data sets from a variety of different geographical locations, represent species diversity, and consider different endpoints. Some disadvantages are that the model only includes 13 PAHs and does not consider alkylated material. There are more than 34 PAHs that should be considered when assessing risk from PAHs (USEPA 2003) and only considering 13 PAHs can significantly underestimate the effect of PAHs. If the 34 PAHs were considered, the comparison against field and laboratory data may have given a different result. Additionally the level of protection for benthic organisms under chronic exposure conditions was not well defined because the toxicity data forming the basis for the TEC evaluation consisted mainly of 10-day survival results for a variety of amphipods.

## **MECHANISTICALLY BASED APPROACHES**

Mechanistically based sediment quality goals (SQGs) were developed as a refinement to empirically based approaches, in part for making SQGs site-specific. That is, they reflect the nature of conditions at a site, and how those conditions affect the bioavailability and toxicity of chemicals. Mechanistic guidelines all incorporate equilibrium partitioning (Di Toro et al. 1991). For PAHs, the toxicity is considered a non-specific mode of action, narcosis.

## ***Background information***

*Carbon-normalized measurements*. The underlying basis for the EqP approach is readily understood by considering the data for midge exposed to kepone in sediment (Adams et al. 1985). The dose-response curves for 14-day midge mortality in relation to dry-weight normalized sediment kepone concentrations as measured in sediments having a wide range of organic carbon contents (fraction organic carbon, f_oc_ = 0.09, 1.5 and 12%) are variable. At 0.5 µg kepone/g dry weight, none of the sediments were acutely toxic (i.e., mortality was comparable to control sediment mortality of <20–25%). At ~3 µg/g, midge mortality increased to nearly 100% in the low f_oc_ sediment while remaining low in the other two sediments, and at about 15 µg/kg, mortality is near 100% in the two lower f_oc_ sediments while remaining low in the high f_oc_ sediment. Finally, at about 80 µg/g, mortality approaches 100% in all three sediments. These sediment toxicity results are typical of what has repeatedly been observed for sediments that vary widely in organic carbon content (see USEPA 2000 and 2005 for additional examples). They clearly demonstrate that the range of sediment chemical concentrations that will be toxic (or safe) over a wide range of sediment types will be sediment-specific.

If these same data are related to either the kepone concentrations in porewater or carbon normalized sediment kepone concentrations, the envelope of previously offset dose-response curves collapses to what is essentially a single curve that provides a consistent response across the full range of sediment types. One cannot infer from the consistency of these results that the route of exposure is via porewater or ingestion of sediment organic carbon; however, because the freely dissolved porewater kepone is likely to be in equilibrium with kepone bound to sediment organic matter, the toxic response is readily correlated to either exposure route (Di Toro et al. 1991). Thus, a definitive determination of the route of exposure is not required.

The utility of the preceding results is derived from the observation of Adams and coworkers that the effects levels in sediment porewater were comparable to effects levels for the same organism in water only exposures (Adams et al. 1985; USEPA 2000). Furthermore, since benthic organisms exhibit a range of sensitivity comparable to water column organisms (Di Toro et al. 1991; USEPA 2005), application of a water quality criterion (WQC) to the porewater will provide a level of protection (LOP) to benthic organisms consistent with the LOP provided to water column organisms. All that remains to evaluate the associated ESB is to relate the bulk sediment concentration to a porewater chemical concentration that will be protective of benthic organisms (i.e., to the WQC) and that it is in equilibrium with.

A simple and readily applicable method is available to relate the porewater chemical concentration to an associated bulk sediment concentration. That is, the organic carbon-normalized sediment concentration (C_SOC_ ~ µg/gOC) is related to the freely dissolved porewater (i.e., non-dissolved-organic-carbon [DOC] complexed) concentration (C_W_ ~ µg/L) via the sediment organic carbon partition coefficient (K_OC_). The relationship is given by:

 (1)

The value of C_SOC_ is computed from the dry weight normalized sediment concentration (CSOC ~ µg/g):

$C_{SOC} = \frac{C_{s}}{f_{oc}}$ (2)

where f_OC_ is the organic carbon content of the sediment (e.g., g_OC_/g). Substitution of the expression for C_SOC_ (Equation 2) and the WQC (for C_W_) into Equation 1, yields a carbon-normalized SQC (SQC_OC_) that provides a level of protection to benthic organisms that is expected to be comparable to what is provided by the WQC to water column organisms:

$SQC_{OC} = K_{OC} WQC$ (4)

*Initial development efforts*. The EqP approach described above was initially applied to three individual PAHs, without considering that PAHs are typically present in environmental settings as mixtures. While the individual SQGs were consistent with the EqP approach, they were inappropriate for use in a typical situation, where PAHs are present as mixtures. Swartz and coworkers provided a significant advance when they directly accounted for the additivity of toxic effects caused by the individual nonionic organic chemicals that compose a mixture (Swartz et al. 1995). Recognition of the non-specific mode of action of PAHs and the need to consider total PAH concentration, as well as the importance of carbon normalization (per the EqP approach, to reflect bioavailability), provided the ∑PAH mixture SQG with a mechanistic tie. However, because the ∑PAH TEC was based on consideration of only 13 commonly measured parent PAHs, rather than the somewhat larger suite of PAHs that may be present and contribute to biological effects (USEPA 2003), and because the relationship between toxicity and the octanol-water partitioning coefficient was based on a relatively small dataset, there was uncertainty associated with its evaluation (Mount et al. 2003). Additionally the level of protection for benthic organisms under chronic exposure conditions was not well defined because the toxicity data forming the basis for the TEC evaluation consisted mainly of 10-day survival results for a variety of amphipods (Swartz et al. 1995).

Swartz (1999) subsequently refined the initial estimates of the total PAH (TPAH, or ∑PAH) guideline in conjunction with efforts to arrive at consensus SQGs for PAHs based on previously proposed SQGs for PAHs in marine sediments. Dry-weight normalized SQGs were expressed in carbon-normalized units by assuming a sediment f_oc_ of 1% (the mean sediment OC in a large database) to facilitate making SQG comparisons with consistent units. When this was done, most of the existing empirical and mechanistically based guidelines were clustered within three groups, depending on their initially intended uses. These groups were referred to as threshold effect concentrations (TECs), median effect concentrations (MECs) and extreme effect concentrations (EECs). The consensus SQGs proposed were simply the arithmetic averages of the SQGs included within each of these three groups (TEC = 290 µg/gOC, MEC = 1,800 µg/gOC, and EEC = 10,000 µg/gOC). Swartz (1999) suggested that the TEC would be the most useful of these because effects due to PAH mixtures were unlikely at concentrations below this lower threshold. Conversely, concentrations exceeding the EEC would likely be associated with obvious adverse impacts. The MEC, which was between the TEC and EEC was judged to be of limited utility because it provided limited discriminatory power regarding the expectation of effects.

*Target lipid model*. The Target Lipid Model (TLM) is another mechanistically based approach that can be used to derive WQC and SQC for nonionic organic chemicals (NIOCs) that exert toxicity via narcosis (Di Toro et al. 2000; Di Toro and McGrath 2000). In accordance with the ∑PAH method, it incorporates EqP concepts and toxic units are summed to account for effects of mixtures. Given the WQC, an SQC_OC_ may then be evaluated in accordance with Equation 4, above. An important factor that differentiates the TLM from other approaches is that it makes use of a species sensitivity distribution (SSD) in a manner analogous to USEPA methods used to derive WQC. This ensures that a TLM-based SQG will be protective of most species and that the level of protection will apply to both acute and chronic effects. The TLM is particularly useful for compounds that exert toxicity by a non-specific mode of action, such as PAHs and other petro-chemical compounds. This is because it provides a way to account for the additive effects caused by the mixture of chemicals present rather than the toxicity of any single chemical. It is for these reasons that USEPA has adopted the TLM as the basis for deriving equilibrium partitioning sediment benchmarks (ESBs) for PAHs that act by a non-specific mode of action (USEPA 2003).

*Equilibrium partitioning-based sediment guidelines (ESBs)*. Several sediment guidelines for individual PAHs using equilibrium partitioning have been proposed and use Equation 4 to compute the guideline value. The difference is in how the WQC were developed and the source of K_OC_. In 1991, USEPA proposed EqP-based sediment guidelines for non-ionic organic chemicals, such as PAHs (USEPA 1991), using the final chronic value derived from existing water quality criteria. If the WQC were developed via the methods of Stephan et al. (1985), then the data were properly vetted and acceptable methods were used. New York State followed this approach and derived EqP-based sediment guidelines using NYS water quality criteria (NYSDEC 1999).

In 2003, USEPA derived ESBs for PAH mixtures that incorporates the TLM and toxic unit approach discussed previously. High quality water-only acute data for single PAHs were compiled and used to derive genus mean acute values, which are equivalent to acute critical target lipid body burdens as defined in the TLM. A species sensitivity distribution was developed, and the 5^th^ percentile was identified as the final acute value. Following the protocols of Stephan et al. (1985), an acute-to-chronic ratio was applied to convert the final acute value to a final chronic value, which is used in Equation 4 to compute a sediment guideline value. ESBs were computed for 34 PAHs including the alkylated homolog series of parent PAHs. Many regulatory agencies have adopted the ESB (Simpson et al. 2013; NYSDEC 2014). Revised Australian and New Zealand sediment guidelines have adopted the ESB approach for sediments where PAHs are considered the dominant chemical of concern (Simpson et al. 2013).

There are many advantages to the ESB approach for PAH mixtures. They were developed from high-quality toxicity databases for acute and chronic values. The chronic values are representative of long-term effects of growth, reproduction, and mortality. The toxicity data covered diverse taxonomic groups. An SSD approach was used which is an accepted approach in risk assessment applications. The method accounts for the suite of 34 PAHs and includes the alkylated homologs, which are excluded from empirical approaches. Furthermore, the method accounts for the covariance of PAHs in sediment by considering the toxicity of the mixture of PAHs. The main disadvantage of the ESB approach is that the partitioning equation assumes that natural organic carbon is the only carbon phase present in the sediment. Other carbon phases (i.e., soot carbon) that have different affinities for the chemical can be present in the sediment. It has been shown that these other phases have a higher affinity for PAHs. As such, ESBs that only consider partitioning to natural organic carbon tend to overestimate bioavailability.

*Maximum permissible concentrations (MPCs)*. MPC – concentration in an environmental compartment at which no effect to be rated as negative is to be expected for an ecosystem’s RIVM derived maximum permissible concentrations (MPC) for 16 individual PAHs (Verbruggen 2012). The MPCs serve to set environmental quality standards, and the SRC can be used to establish intervention values. These are updated environmental risk limits first proposed in 2001 (Verbruggen et al. 2001). Data on toxicological effects for PAHs in water, sediment and soil were compiled and thoroughly evaluated for quality and reliability. The limits were derived based on a recognized and established framework in the Netherlands.

For sediments, preference was given to available chronic toxicity data. The chronic data were evaluated and the lowest reliable value was selected as the MPC adjusted by an appropriate assessment factor. The assessment factors are based on the number of reliable chronic data available as recommended in the Technical Guidance Document (TGD) (European Commission 2003) and range in value from 1 to 1,000. Based on the number of reliable chronic data, the MPC may need to be compared to a value computed by equilibrium partitioning where the water effect concentration was determine in Verbruggen (2012). If no sediment data were available, then EqP is used to establish the MPC. The MPC for sediments are converted to dry weight basis using the Dutch standard of 10% organic carbon for sediments.

The advantages of the MPC approach are that it uses high-quality water and sediment toxicity data, it considers bioavailability, and it uses a recognized and standardized method for computing environmental risk limits. In addition, the protection afforded is appropriate for protection of the benthic organisms from adverse effects. The disadvantages are the use of arbitrary assessments factors based on the quantity of reliable data available and the assumed 10% organic carbon standard, which is high and could result in conservative risk limits.

Sediment quality criteria for 16 PAHs based on a classification system for protection of sediments in Norway have been proposed but not adopted (Bakke et al. 2010). In that system, concentrations of contaminants below their respective chronic probable-no-effect concentration (PNEC) were considered to have no adverse impacts on the benthic community. The sediment chronic PNEC was derived with similar methods as Verbruggen (2012) such that sediment toxicity data were used if available or established from a water PNEC and EqP relationship. The methods for computing a PNEC followed TGD protocols. The difference is that for these sediments an organic carbon concentration of 1% was used. The toxicity data in the development of the PNEC values were not provided, and as such, it could not be verified whether the TGD methods were strictly followed. However, they are included for comparison and have the same advantages and disadvantages as the MPC values.

## **REFERENCES**

Adams WJ, Kimerle RA, Mosher RG. 1985. Aquatic safety assessment of chemicals sorbed to sediments. In: Cardwell RD, Purdy R, Bahner RC, editors. Aquatic Toxicology and Hazard Assessment: Seventh Symposium, Special Technical Publication STP 854, American Society for Testing and Materials (ASTM), Philadelphia, PA, pp. 429–453.

Arp HPH, Hale SE, Krusa ME, Cornelissen GC, Grabanski CB, Miller DJ, Hawthorne SB. 2015. Review of polyoxymethylene passive sampling methods for quantifying freely dissolved porewater concentrations of hydrophobic organic contaminants. *Environ Toxicol Chem* 34:710–720.

Bakke T, Kallqvist T, Ruus A, Breedveld GD, Hylland K. 2010. Development of sediment quality criteria in Norway. *J Soils Sediments* 10:172–178.

Barrick R, Becker S, Brown L, Beller H, Pastorok R. 1988. Sediment quality values refinement: Volume 1. 1988 update and evaluation of Puget Sound AET. Final Report submitted by PTI to Tetra Tech for the Puget Sound Estuary Program EPA Contract N. 68-01-4341.

Buchman MF. 1999. NOAA screening quick reference tables: NOAA HAZMAT Report 99–1. Seattle: Coastal Protection and Restoration Division. p. 12.

[CCME] Canadian Council of Ministers of the Environment. 1999. Protocol for the derivation of Canadian Sediment quality guidelines for the protection of aquatic life. Ottawa, Ontario, Canada. 27 pages.

Di Toro DM, Zarba CS, Hansen DJ, Berry WJ, Schwartz RC, Cowan CW, Pavlou SP, Allen HE, Thomas NA, Paquin PR. 1991. Technical basis for establishing sediment quality criteria for nonionic organic chemicals by using equilibrium partitioning. *Environ Toxicol Chem* 10:1541–1583.

Di Toro DM, McGrath JA, Hansen DJ. 2000. Technical basis for narcotic chemicals and PAH criteria. I. Water and tissue. *Environ Toxicol Chem* 19:1951–1970.

Di Toro DM, McGrath JA. 2000. Technical basis for narcotic chemicals and polycyclic aromatic hydrocarbon criteria. II. Mixtures and sediments. *Environ Toxicol Chem* 19:1971–1982.

European Commission. 2003. Technical Guidance Document in support of Commission Directive 93/67/EEC on Risk Assessment for new notified substances, Commission Regulation (EC) No 1488/94 on Risk Assessment for existing substances and Directive 98/9/EC of the European Parliament and of the Council concerning the placing of biocidal products on the market. Part II. Ispra, Italy: Joint Research Centre, Institute for Health and Consumer Protection, European Chemicals Bureau (ECB). Draft report, final version R323.

Field LJ, MacDonald DD, Norton SB, Ingersoll CG, Severn CG, Smorong D, Lindskoog R. 2002. Predicting amphipod toxicity from sediment chemistry using logistic regression models. *Environ Toxicol Chem* 21:1993–2005.

Gries TH, Waldow KH. 1996. Progress re-evaluating Puget Sound Apparent Effects Thresholds (AETS) Volume 1: 1994 Amphipod and echinoderm larval AET. Washington Department of Ecology, Washington, USA.

Ingersoll CG, Haverland PS, Brunson EL, Canfield TJ, Dwyer FJ, Henke CE, Kemble NE, Mount DR, Fox RG. 1996. Calculation and evaluation of sediment effects concentrations for the amphipod *Hyalella azteca* and the midge Chironomus riparius. *J Great Lakes Res* 22:602–623.

Long ER, Morgan LG. 1991. The potential for biological effects of sediment-sorbed contaminants tested in the National Status and Trends Program. Technical memorandum NOS OMA 52. National Oceanic and Atmospheric Agency. Rockville, MD, USA.

Long ER, MacDonald DD, Smith FL, Calder FD. 1995. Incidence of adverse biological effects within ranges of chemical concentrations in marine and estuarine sediments. *Environ Manag* 19:81–97.

MacDonald DD. 1992. Development of an approach to the assessment of sediment quality in Florida coastal waters. Prepared by MacDonald Environmental Sciences, Ltd. Ladysmith, BC. Prepared for Florida Department of Environmental Regulation. Tallahassee, FL.

MacDonald DD, Carr RS, Calder FD, Long ER, Ingersoll CG. 1996. Development and evaluation of sediment quality guidelines for Florida coastal waters. *Ecotoxicology* 5:253–278.

MacDonald DD, Ingersoll CG, Berger TA. 2000. Development and evaluation of consensus-based sediment quality guidelines for freshwater ecosystems. *Arch Environ Contam Toxicol* 39:20–31.

Menchaca I, Rodriguez JG, Borja A, Belzunce-Segarra MJ, Franco J, Garmendia JM, Larreta J. 2014. Determination of polychlorinated biphenyl and polycyclic aromatic hydrocarbon marine regional sediment quality guidelines within the European Water Framework Directive. Chem Ecol 30:693–700.

Mount DR, Ingersoll CG, McGrath JA. 2003. Approaches to developing sediment quality guidelines for PAHs. In: Douben, PET, editor. PAHs: An Ecotoxicological Perspective. Chichester, England: John Wiley & Sons, pp. 331–355.

Neff JM, Bean DJ, Cornaby BW, Vaga RM, Gulbransen TC, Scanlon JA. 1986. Sediment quality criteria methodology and validation: Calculation of screening level concentrations from field data. Work Assignment 56, Task IV. Battelle Institute. Prepared for U.S. Environmental Protection Agency, Washington, DC, 225 pp.

Neff JM, Word JQ, Gulbranson TC. 1987. Recalculation of screening level concentrations of non-polar organic contaminants in marine sediments. Final Report. US Environmental Protection Agency, Washington, DC, USA.

NYSDEC. 1999. Screening and assessment of contaminated sediment. New York State Department of Environmental Conservation, Division of Fish, Wildlife and Marine Resources, Albany, NY 39pp.

NYSDEC. 2014. Screening and assessment of contaminated sediment. New York State Department of Environmental Conservation, Division of Fish, Wildlife and Marine Resources, Albany, NY 99pp.

Persaud D, Jaagumagi R, Hayton A. 1993. Guidelines for the protection and management of aquatic sediment quality in Ontario. Water Resources Branch, Ontario Ministry of the Environment, Toronto Canada.

Simpson SL, Batley GB, Chariton AA. 2013. Revision of the ANZECC/ARMCANZ sediment quality guidelines. CSIRO Land and Water Science Report 09/07. CSIRO Land and Water.

Smith SL, MacDonald DD, Keenleyside KA, Ingersoll CG, Field LJ. 1996. A preliminary evaluation of sediment quality assessment values for freshwater ecosystems. *J Great Lakes Res* 22:624–638.

Stephan CE, Mount DI, Hansen DJ, Gentile JH, Chapman GA, Brungs WA. January 1985. Guidelines for deriving numerical National water quality criteria for the protection of aquatic organisms and their uses. USEPA Office of Research and Development, Environmental Research Laboratories: Duluth, Minnesota; Narragansett, Rhode Island, and Corvallis, Oregon, PB85-227049, 98 pp.

Swartz RC, Schults DW, Ozretich RJ, Lamberson JO, Cole FA, DeWitt TH, Redmond MS, Ferraro SP. 1995. ∑PAH: A model to predict the toxicity of polynuclear aromatic hydrocarbon mixtures in field-collected sediments. *Environ Toxicol Chem* 14(11):1977–1987.

Swartz RC. 1999. Consensus sediment quality guidelines for polycyclic aromatic hydrocarbon mixtures. *Environ Toxicol Chem* 18:780–787.

USEPA. 1991. Proposed technical basis for establishing sediment quality criteria for nonionic organic chemicals using equilibrium partitioning. U.S. EPA Office of Science and Technology, Washington, DC, USA.

USEPA. 2000. Equilibrium partitioning sediment guidelines (ESGs) for the protection of benthic organisms: PAH mixtures (draft). U.S. Environmental Protection Agency Office of Water, Washington, DC, USA.

USEPA. 2003. Procedures for the derivation of equilibrium partitioning sediment benchmarks (ESBs) for the protection of benthic organisms: PAH mixtures. U.S. Environmental Protection Agency, Washington, DC, EPA-600-R-02-013.

USEPA. 2005. Procedures for the derivation of equilibrium partitioning sediment benchmarks (ESBs) for the protection of benthic organisms: Metal mixtures (cadmium, copper, lead, nickel, silver, and zinc), U.S. Environmental Protection Agency, Washington, DC, EPA-600-R-02-011.

Verbruggen EMJ. 2012. Environmental risk limits for polycyclic aromatic hydrocarbons (PAHs) for direct aquatic, benthic and terrestrial toxicity. Bilthoven, The Netherlands: National Institute of Public Health and the Environment. RIVM report 607711007.

Verbruggen EMJ, Posthumus R, Van Wezel AP. 2001 Ecotoxicological serious risk concentrations for soil, sediment and (ground)water: updated proposals for first series of compounds. Bilthoven, The Netherlands: National Institute of Public Health and the Environment. RIVM report 711701020.

[WDE] Washington State Department of Ecology. 2015. Sediment cleanup users manual II. Guidance for implementing the cleanup provisions of the sediment management standards, Chapter 173-204 WAC. Publication No. 12-09-057. Washington, USA.

[WDNR] Wisconsin Department of Natural Resources. 2003. Consensus-based sediment quality guidelines. Recommendations for use and application. Interim Guidance. Madison, WI.

| **Table S1. Guidelines Reviewed for PAHs** | | | | | | |
| --- | --- | --- | --- | --- | --- | --- |
| Guideline | Guideline Type | Objective | Data Type | Freshwater / Marine | Biological and Chemical Data | Reference |
| Effects Range Low (ERL):  sediment concentration below which effects are rarely observed | Empirical | Protective | National databases; field data; laboratory spiked data; various sediment quality guidelines | Mixed | paired data; mixed endpoints (mortality, abundance, growth, avoidance, burrowing time, etc.), mixed species; measured and nominal chemistry | Long and Morgan, 1990; Long et al. 1995 |
| Effects Range Median (ERM):  sediment concentration above which effects are frequently or always observed or predicted | Empirical | Predictive | National databases; field data; laboratory spiked data; various sediment quality guidelines | Mixed | paired data; mixed endpoints (mortality, abundance, growth, avoidance, burrowing time, etc.), mixed species; measured and nominal chemistry | Long and Morgan, 1990; Long et al. 1995 |
| Apparent Effects Threshold (AET):  sediment concentration of a contaminant above which statistically significant adverse effects for a particular biological indicator are always expected relative to controls | Empirical | Predictive | Puget Sound survey data; bioassays | Marine | paired data; AETs for specific biological effects (e.g., amphipods, oyster, bacteria) and lowest AET taken to be AET-L | Barrick et al. 1988; Gries and Waldow 1996 |
| Threshold Effect Concentration (TEC):  sediment concentration below which adverse effects are not expected to occur | Empirical | Protective | Various databases | Freshwater | paired data; mixed endpoints; mixed species | MacDonald et al. 2000; Swartz 1999 |
| Probable Effect Concentration (PEC):  sediment concentration above which adverse above which effects are expected to occur more often than not | Empirical | Predictive | Various databases | Freshwater | paired data; mixed endpoints; mixed species | MacDonald et al. 2000; Swartz 1999 |
| Threshold Effects Level (TEL):  sediment concentration below which effects are rarely observed | Empirical | Protective | Florida coastal waters;  North American waters; | Marine Freshwater | paired data/mixed endpoints/mixed species^ab^; individual endpoints for *Hyalella* and *Chironomus*^c^ | MacDonald et al. 1996 Smith et al. 1996 Ingersoll et al. 1996 |
| Probable Effects Level (PEL):  sediment concentration above which toxic effects are frequently observed | Empirical | Predictive | Florida coastal waters;  North American waters | Marine Freshwater | paired data/ mixed endpoints/mixed species^ab^; individual endpoints for *Hyalella* and *Chironomus*^c^ | MacDonald et al. 1996 Smith et al. 1996 Ingersoll et al. 1996 |
| Midpoint Effect Concentration (MEC):  sediment concentration midway between the TEC and PEC | Empirical | Predictive | Various databases | Freshwater | paired data; mixed endpoints; mixed species | WDNR 2003 |
| Screening Level Concentration (SLC):  Concentration of a nonpolar organic contaminant in sediment which, if exceeded, could lead to environmental degradation and therefore warrants further investigation | Empirical | Predictive | Various databases | Freshwater and Saltwater | Sediment concentrations for various locations; variety of species determined for their presence | Neff et al. 1986 |
| Lowest Effect Level (LEL):  Sediment concentration of a compound that can be tolerated by the majority of benthic species | Empirical | Protective | Various databases in Ontario | Freshwater | Sediment concentrations for various locations; variety of species determined for their presence | Persaud et al. 1993 |
| Severe Effect Level (SEL):  Sediment concentration of a compound that would be detrimental to the majority of benthic species. | Empirical | Predictive | Various databases in Ontario | Freshwater | Sediment concentrations for various locations; variety of species determined for their presence | Persaud et al. 1993 |
| Interim Sediment Quality Guideline (ISQG):  Sediment concentration below which adverse effects are rarely observed | Empirical | Protective | Uses various data available from North America | Freshwater and Saltwater values | paired data; different species; different endpoints | CCME 1999 |
| Sediment Cleanup Objective (SCO):  Lower end of a range of chemical concentrations or biological effects levels used to establish sediment cleanup level | Empirical | Protective | Regional databases | Freshwater/ Marine | paired chemistry and biological data; different species and endpoints | WDE  2015 |
| Cleanup Screening Level (CSL):  Maximum chemical concentration or biological effects level allowed as a sediment cleanup level | Empirical | Predictive | Regional databases | Freshwater/ Marine | paired chemistry and biological data; different species and endpoints | WDE  2015 |
| T20 and T50:  Chemical concentration corresponding to a 20 or 50% probability of observing sediment toxicity | Empirical | Protective (T20) or predictive (T50) | Toxicity database throughout North America (Atlantic, Pacific, Gulf coasts) | Marine | paired sediment chemistry and biological effects; 10-day amphipod toxicity tests | Field et al. 2002 |
| Equilibrium Partitioning Sediment Benchmarks (ESBs):  Computes a Final chronic value for each PAH that should protect 95% of tested species | Mechanistic | Protective | Water-only toxicity data | Freshwater and marine | different species; endpoints growth, reproduction of mortality | USEPA 1991, 2003 |
| Serious Risk Concentration (SRC):  Sediment is considered seriously contaminated if SRC is exceeded | Mechanistic | Predictive | Water-only or sediment toxicity data | Freshwater and marine | Toxicity test or QSARs based on log K_OW_ | Verbruggen et al. 2001, Verbruggen 2012 |
| Maximum Permissible Concentration (MPC):  The concentration that has no effect as negative for ecosystems | Mechanistic | Protective | Water-only or sediment toxicity data | Freshwater and marine | Toxicity test or QSARs based on log K_OW_ | Verbruggen 2012 |
| Upper Effect Threshold (UET):  sediment concentration of a contaminant above which statistically significant adverse effects for a particular biological indicator are always expected relative to controls | Empirical | Protective |  |  |  | Buchman 1999 |

| **Table S2. Protective Guidelines Accuracy Test. 187 Samples with Toxicity Data (Arp et al. 2015)** | | |
| --- | --- | --- |
|  | | |
|  | Not Toxic | Toxic |
| < Guideline | # Correct | # False Negative |
| > Guideline | # False Positive | # Correct |
|  |  |  |
| ERL | Not Toxic | Toxic |
|  | 28 | 2 |
|  | 119 | 38 |
| LEL | Not Toxic | Toxic |
|  | 28 | 2 |
|  | 119 | 38 |
| TEL | Not Toxic | Toxic |
|  | 7 | 0 |
|  | 140 | 40 |
| TEC | Not Toxic | Toxic |
|  | 18 | 1 |
|  | 129 | 39 |
| PNEC | Not Toxic | Toxic |
|  | 23 | 12 |
|  | 124 | 28 |
| ESB | Not Toxic | Toxic |
|  | 29 | 1 |
|  | 118 | 39 |
| ERM | Not Toxic | Toxic |
|  | 86 | 4 |
|  | 61 | 36 |
| SEL | Not Toxic | Toxic |
|  | 110 | 8 |
|  | 37 | 32 |
| PEL | Not Toxic | Toxic |
|  | 27 | 2 |
|  | 120 | 38 |
| PEC | Not Toxic | Toxic |
|  | 66 | 3 |
|  | 81 | 37 |


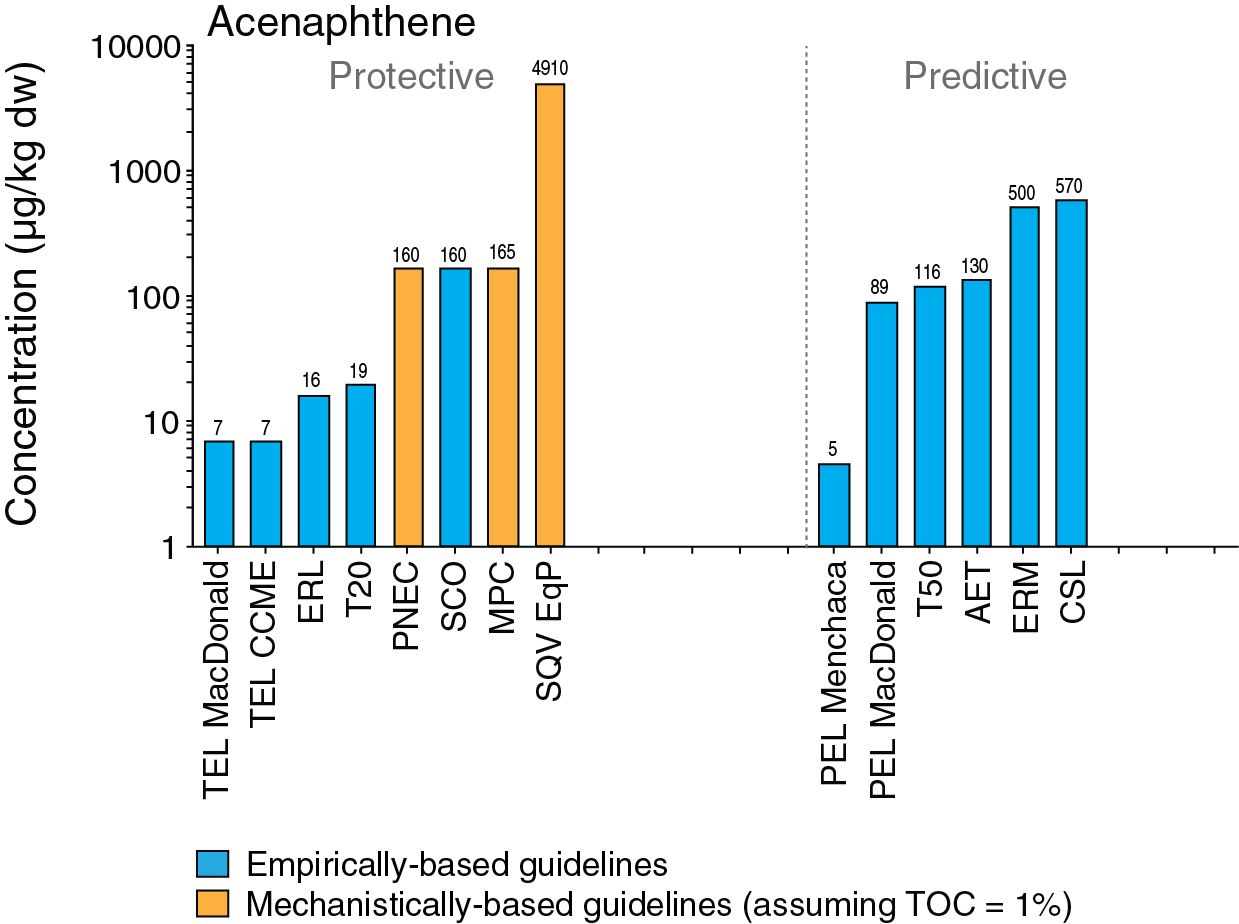


Figure S1. Comparison of guidelines for acenaphthene.


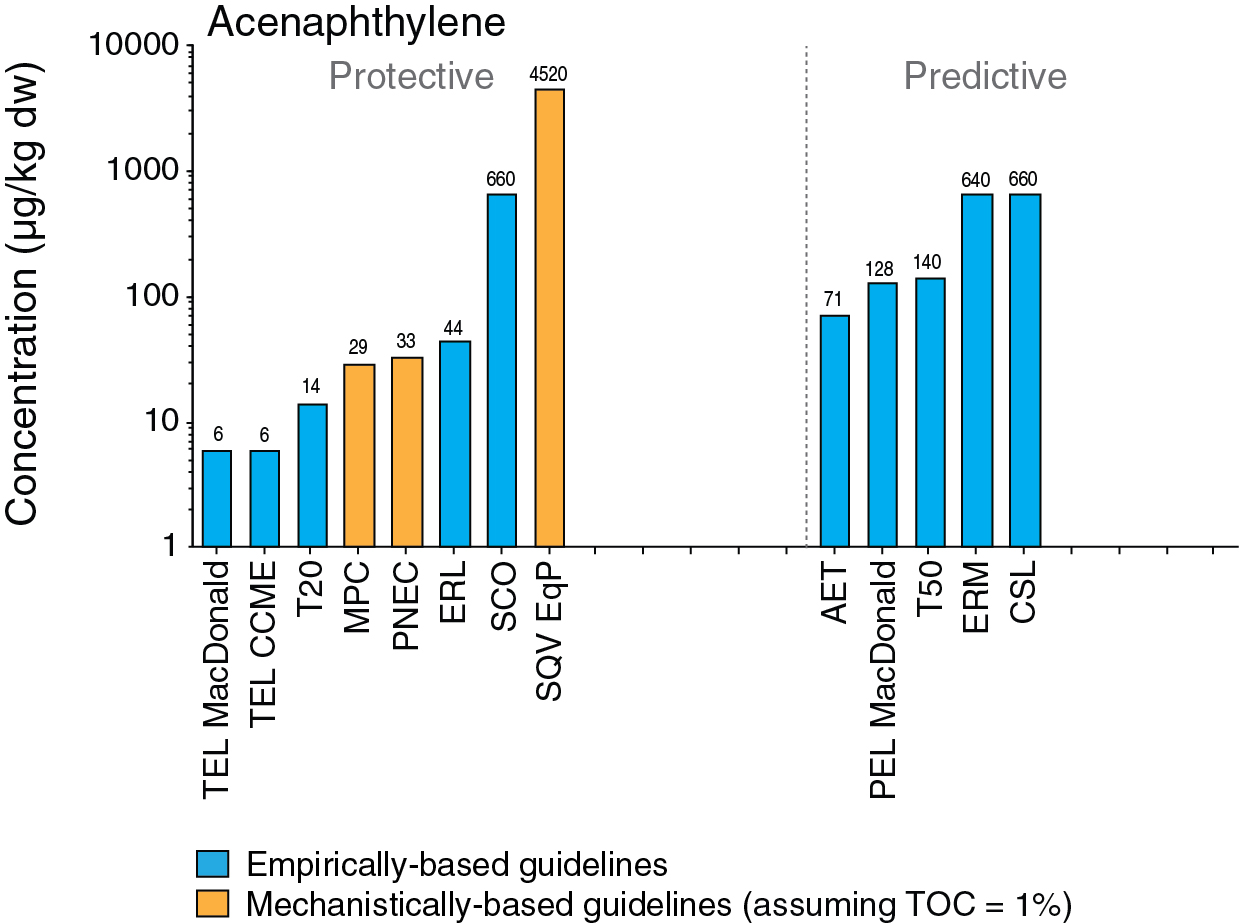


Figure S2. Comparison of guidelines for acenaphthylene.


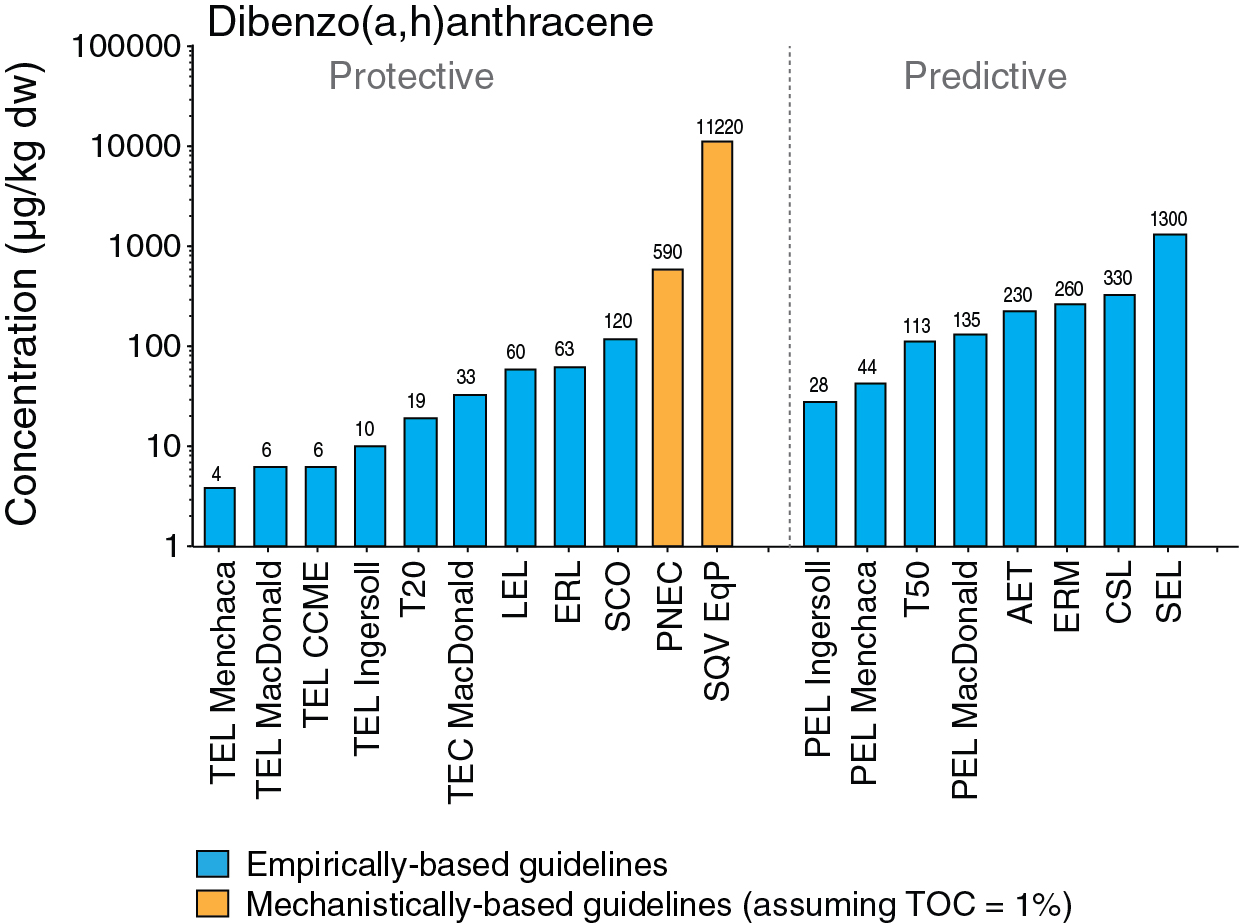


Figure S3. Comparison of guidelines for dibenzo(a,h)anthracene


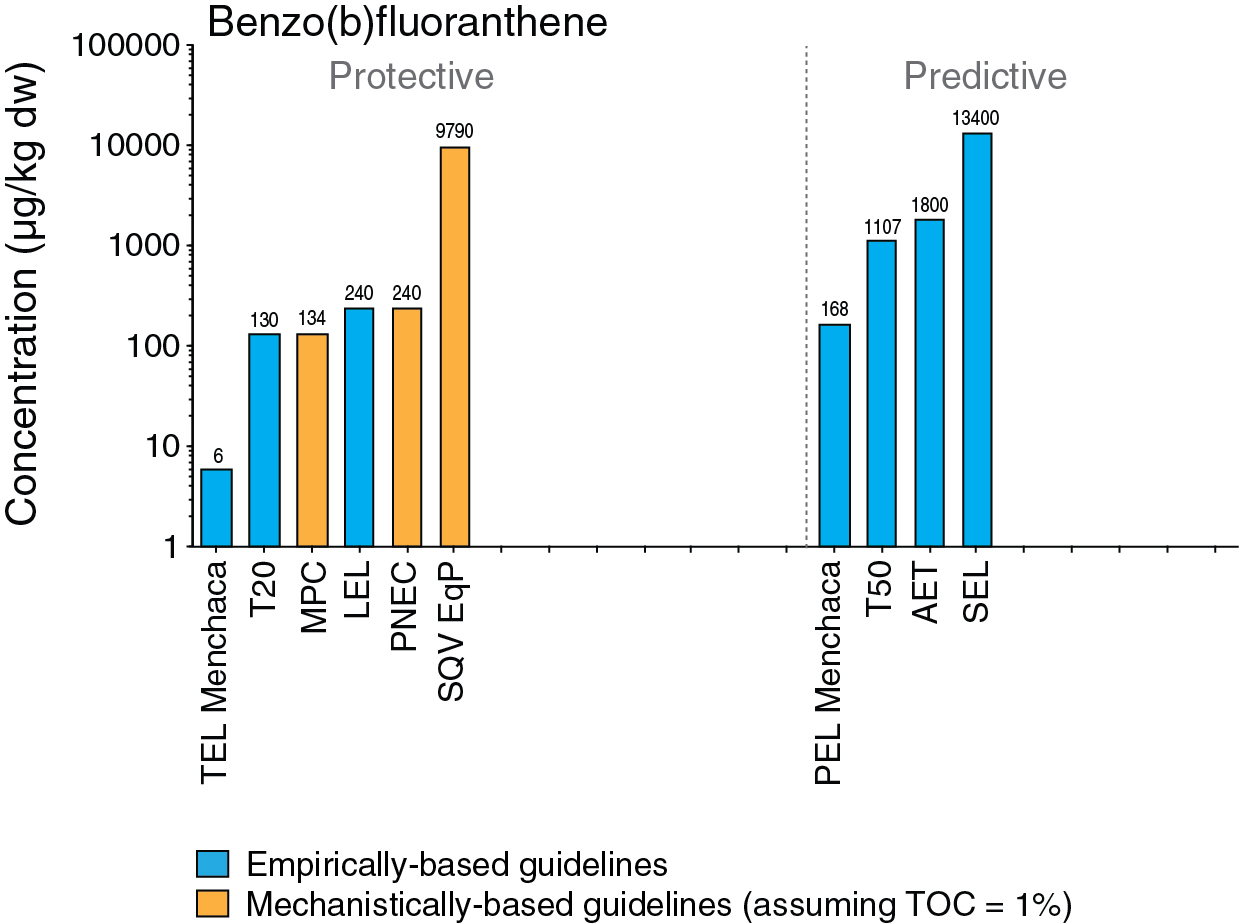


Figure S4. Comparison of guidelines for benzo(b)fluoranthene.


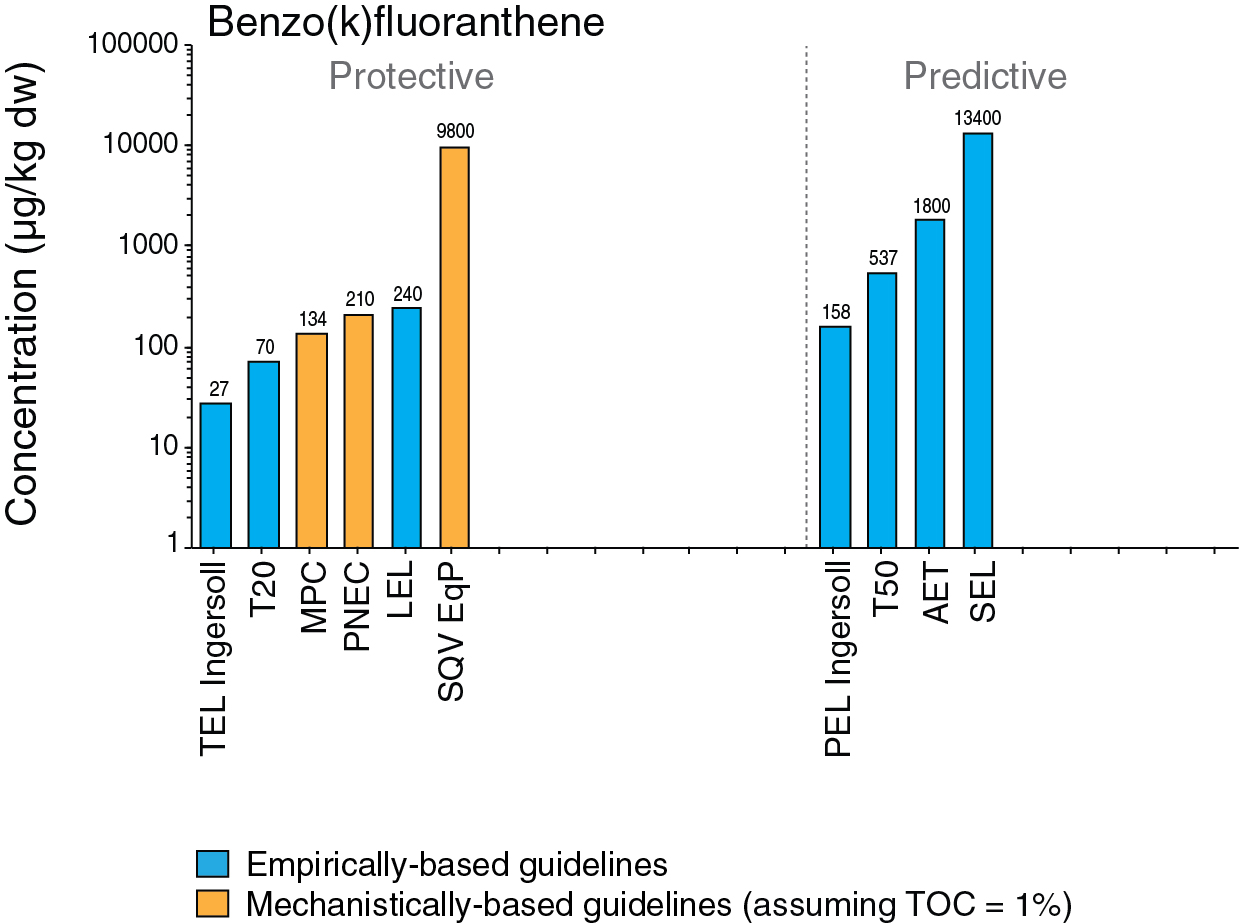


Figure S5. Comparison of guidelines for benzo(k)fluoranthene.


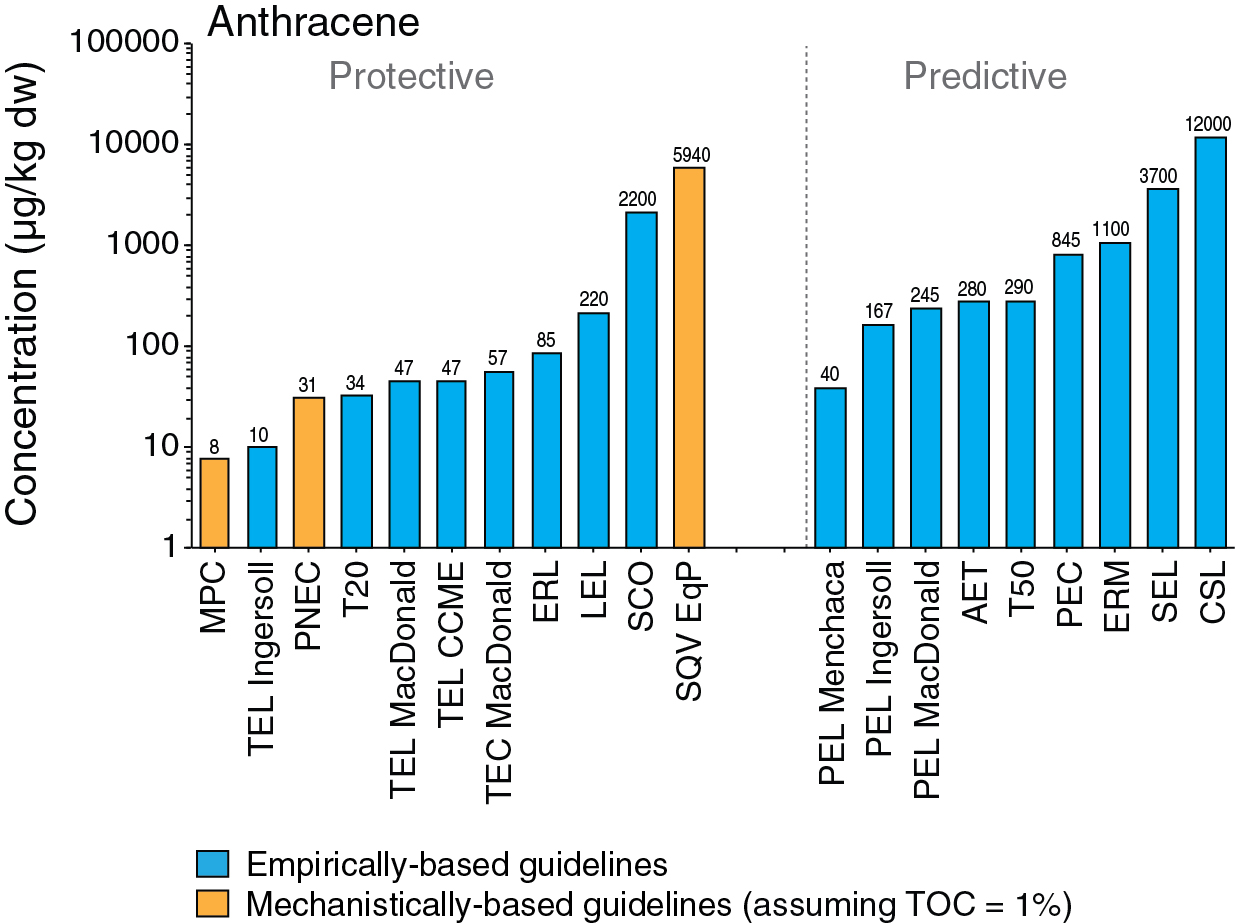


Figure S6. Comparison of guidelines for anthracene.


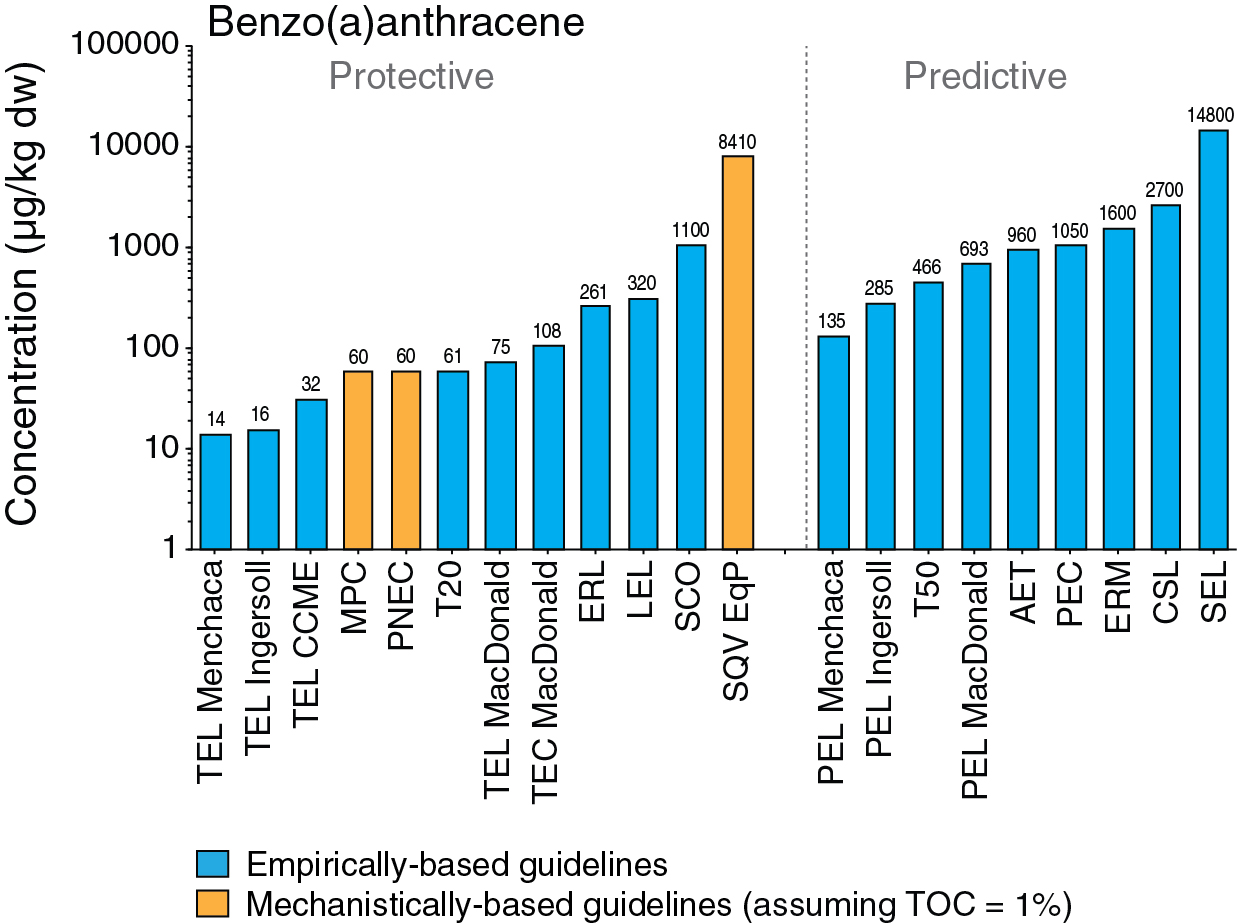


Figure S7. Comparison of guidelines for benzo(a)anthracene.


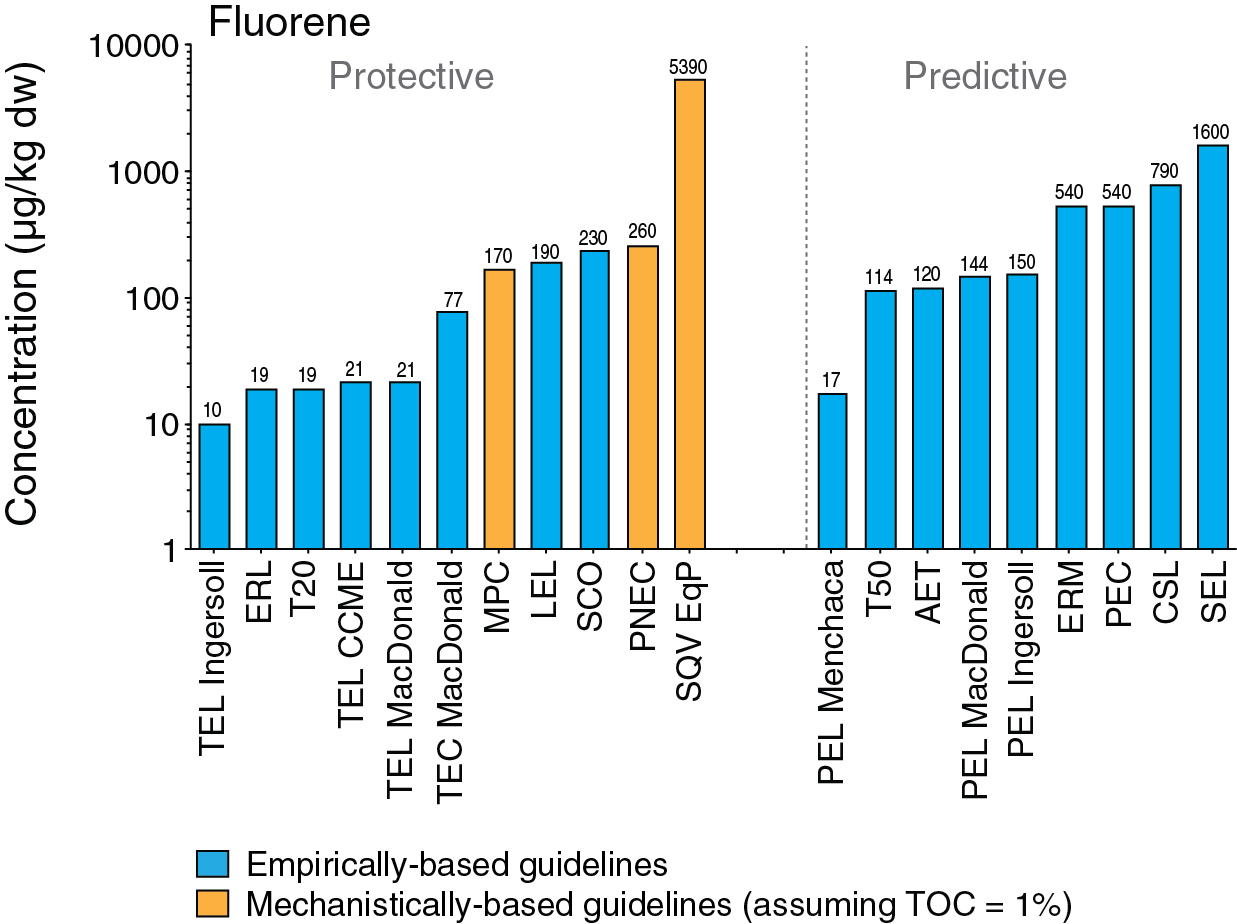


Figure S8. Comparison of guidelines for fluorene.


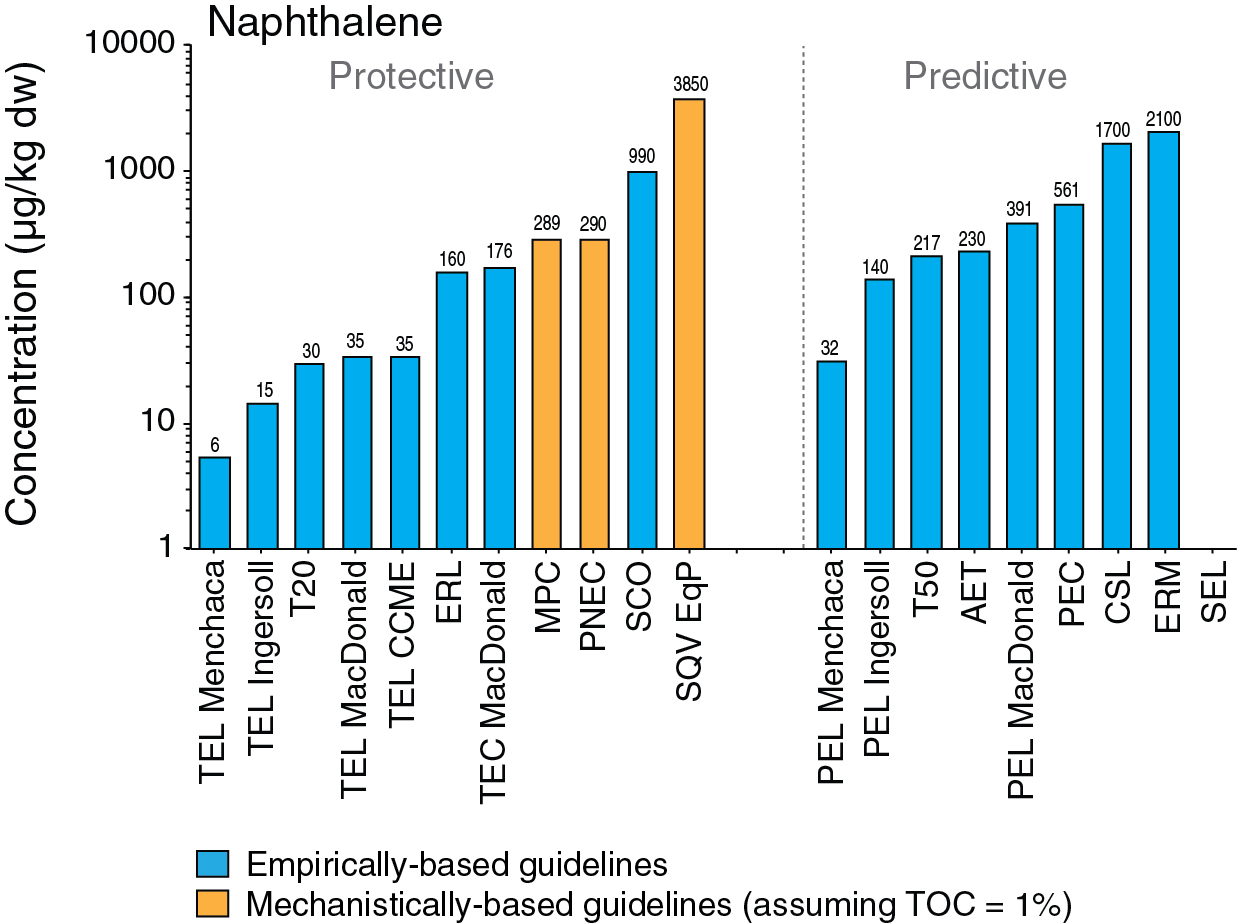


Figure S9. Comparison of guidelines for naphthalene.


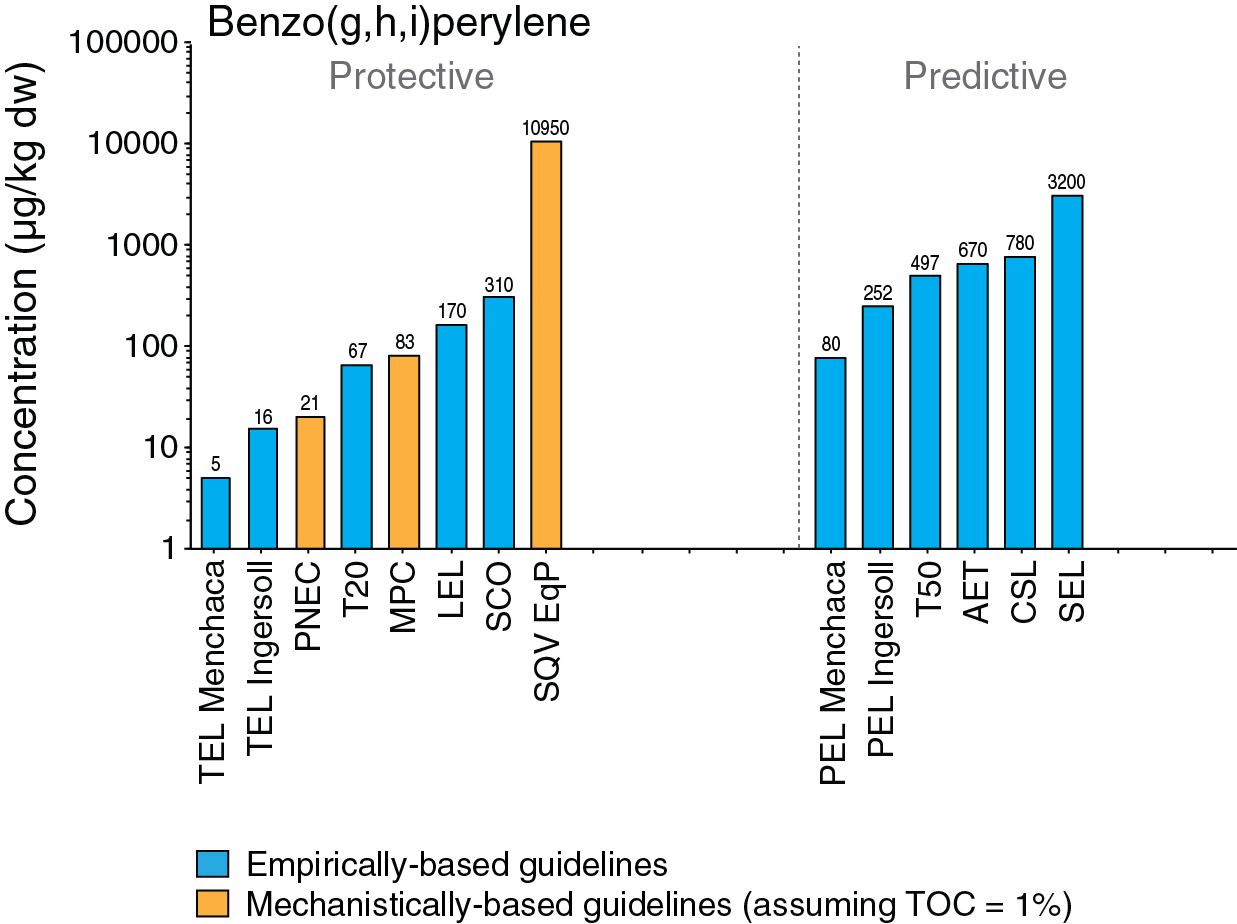


Figure S10. Comparison of guidelines for benzo(g,h,i)perylene


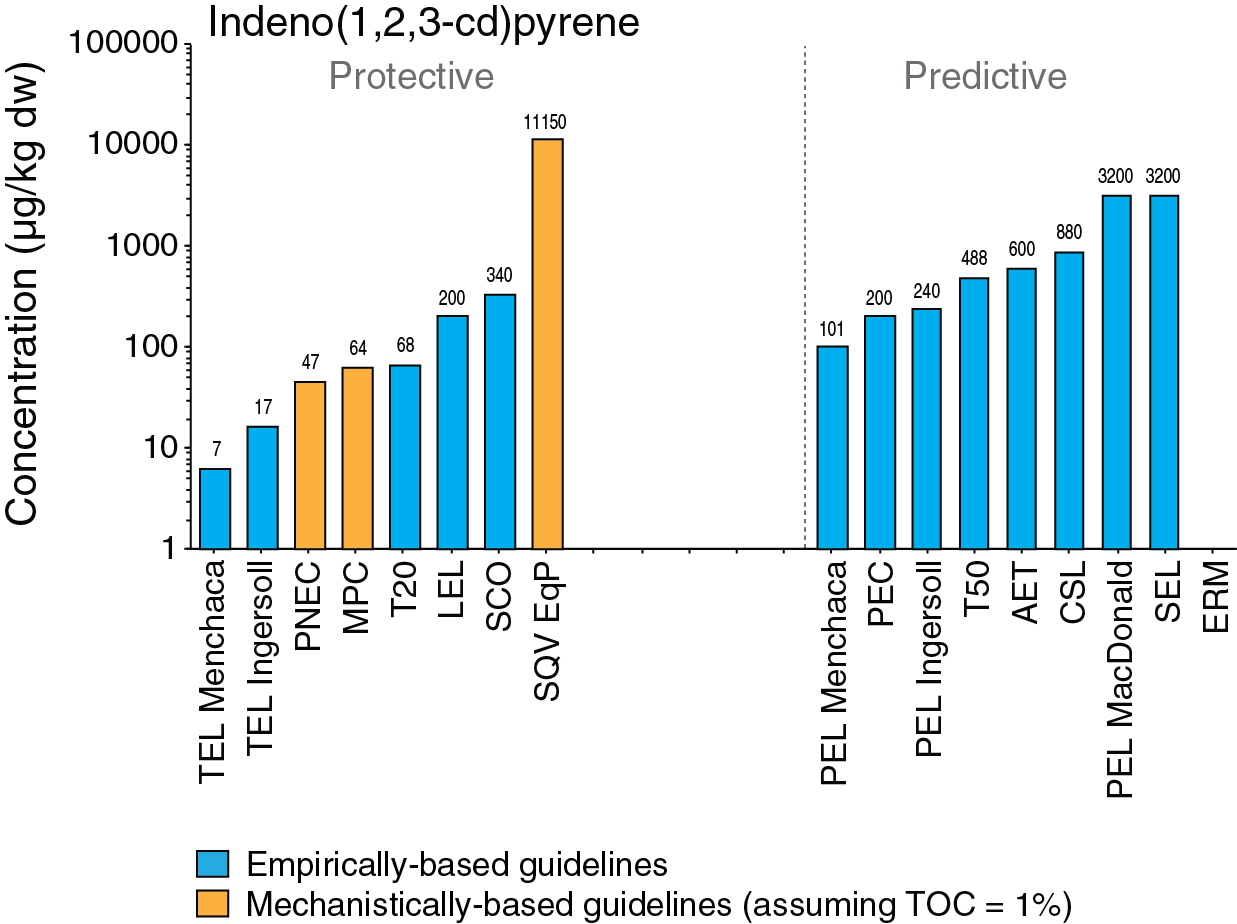


Figure S11. Comparison of guidelines for indeno(1,2,3-cd)pyrene.


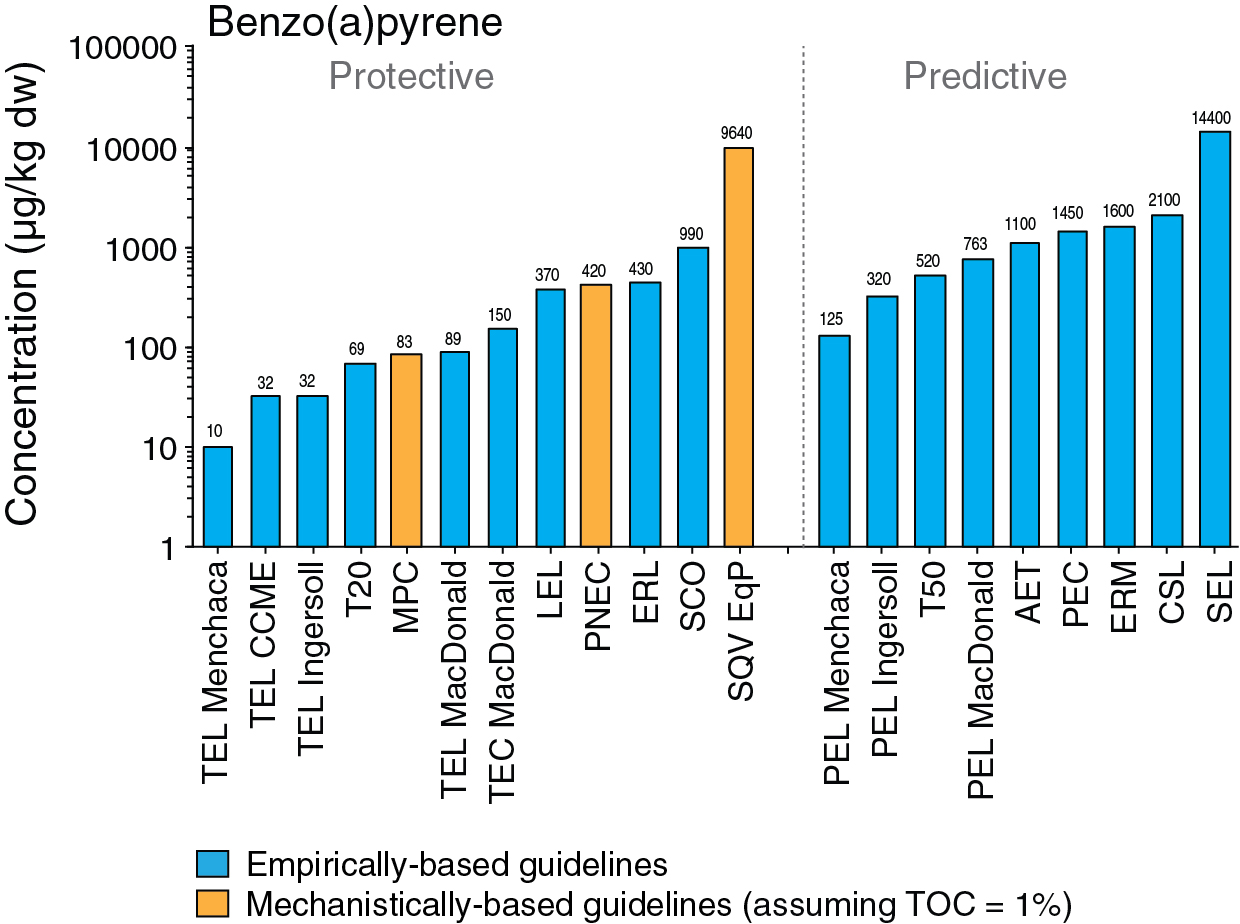


Figure S12. Comparison of guidelines for benzo(a)pyrene.


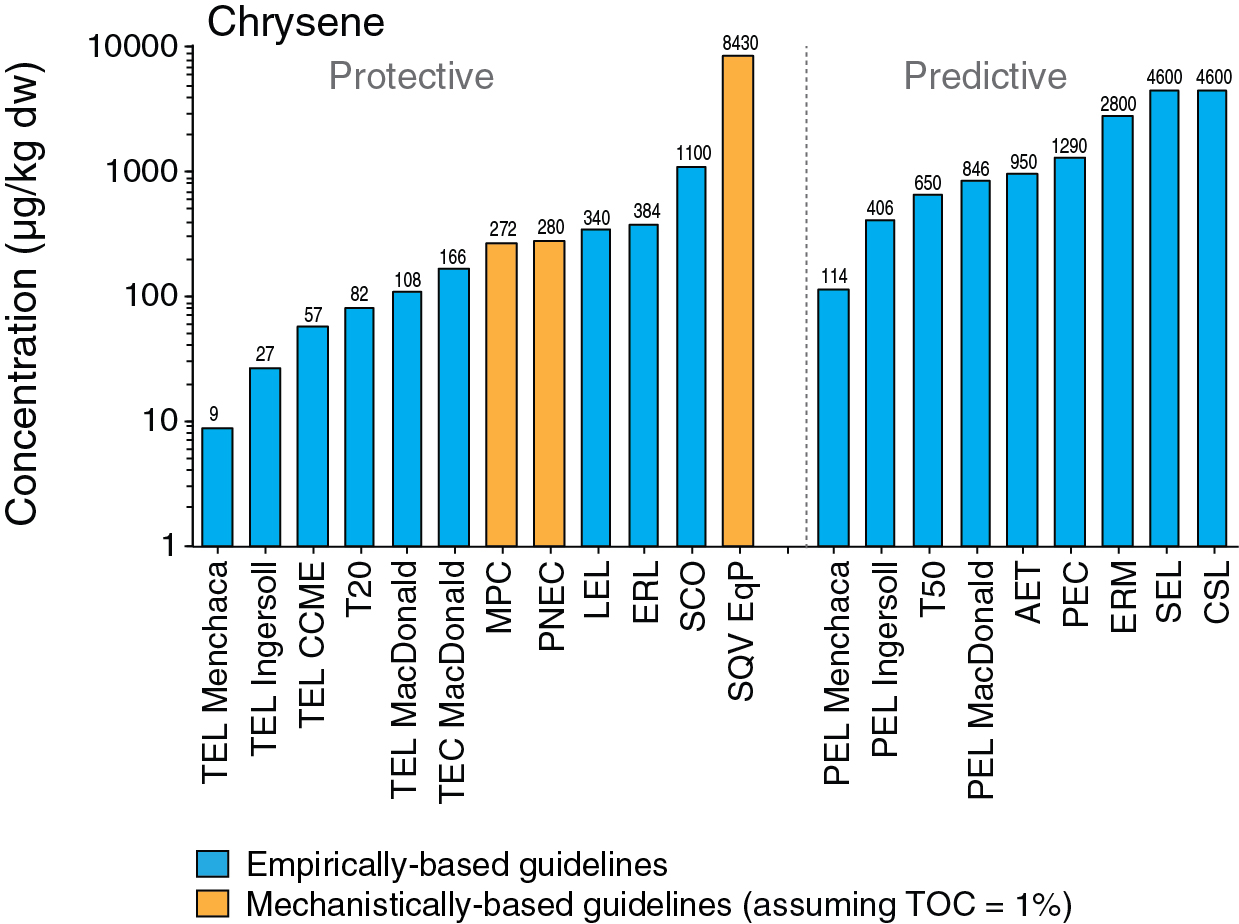


Figure S13. Comparison of guidelines for chrysene.


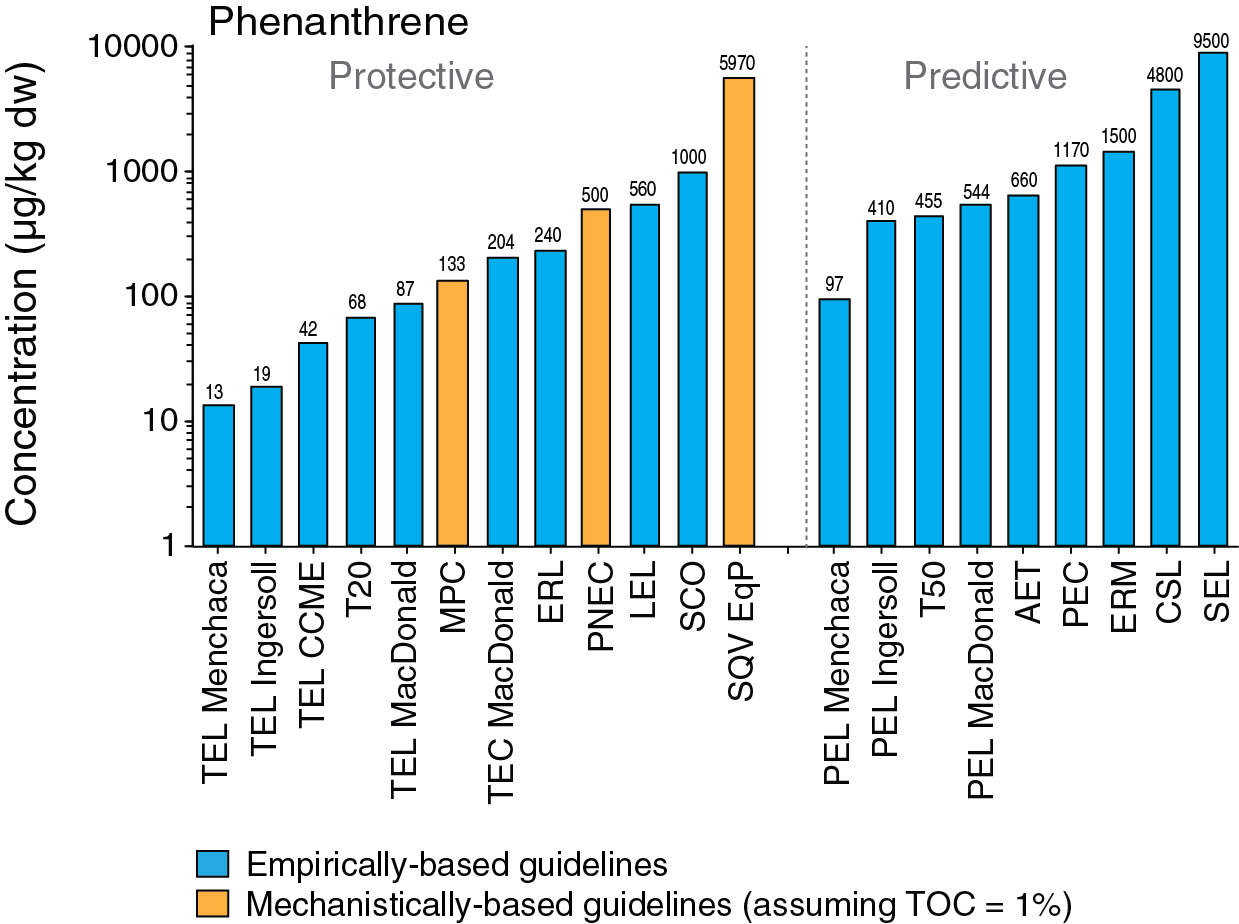


Figure S14. Comparison of guidelines for phenanthrene.


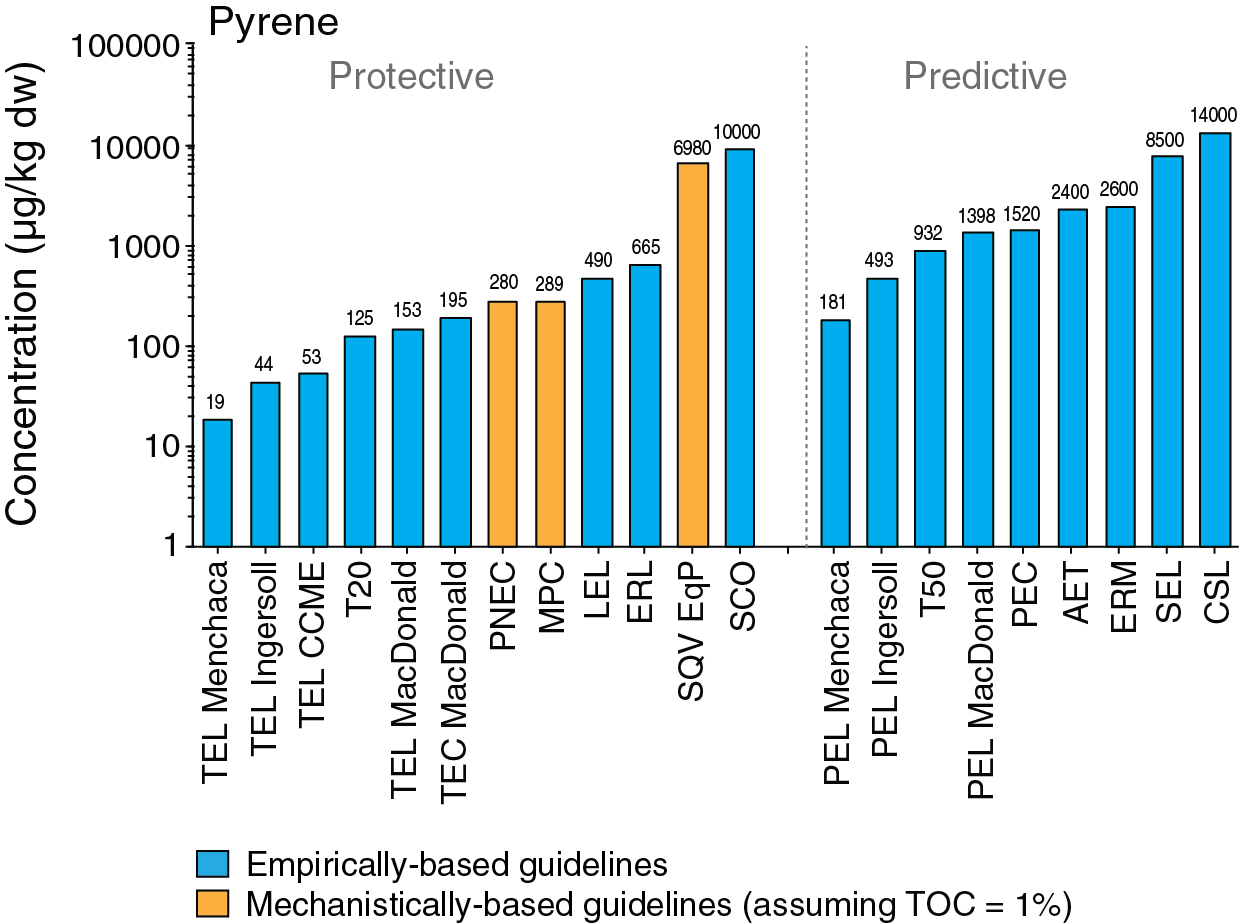


Figure S15. Comparison of guidelines for pyrene.


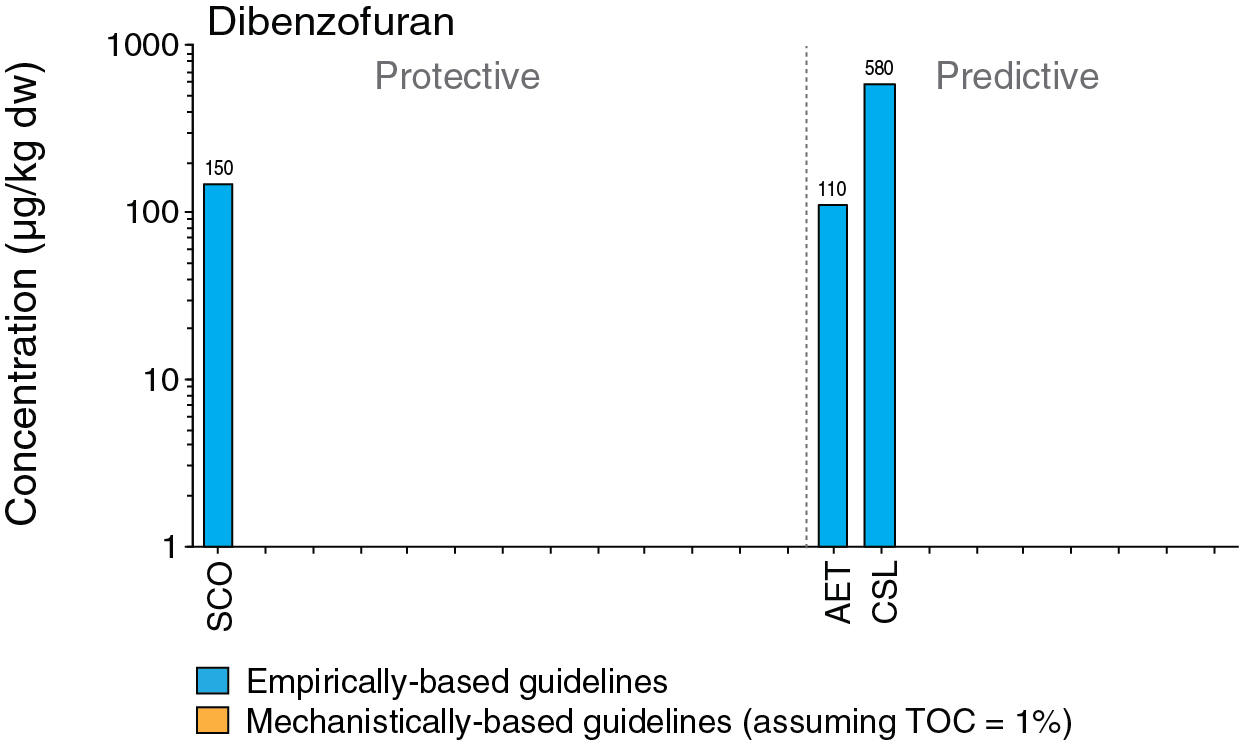


Figure S16. Comparison of guidelines for dibenzofuran.


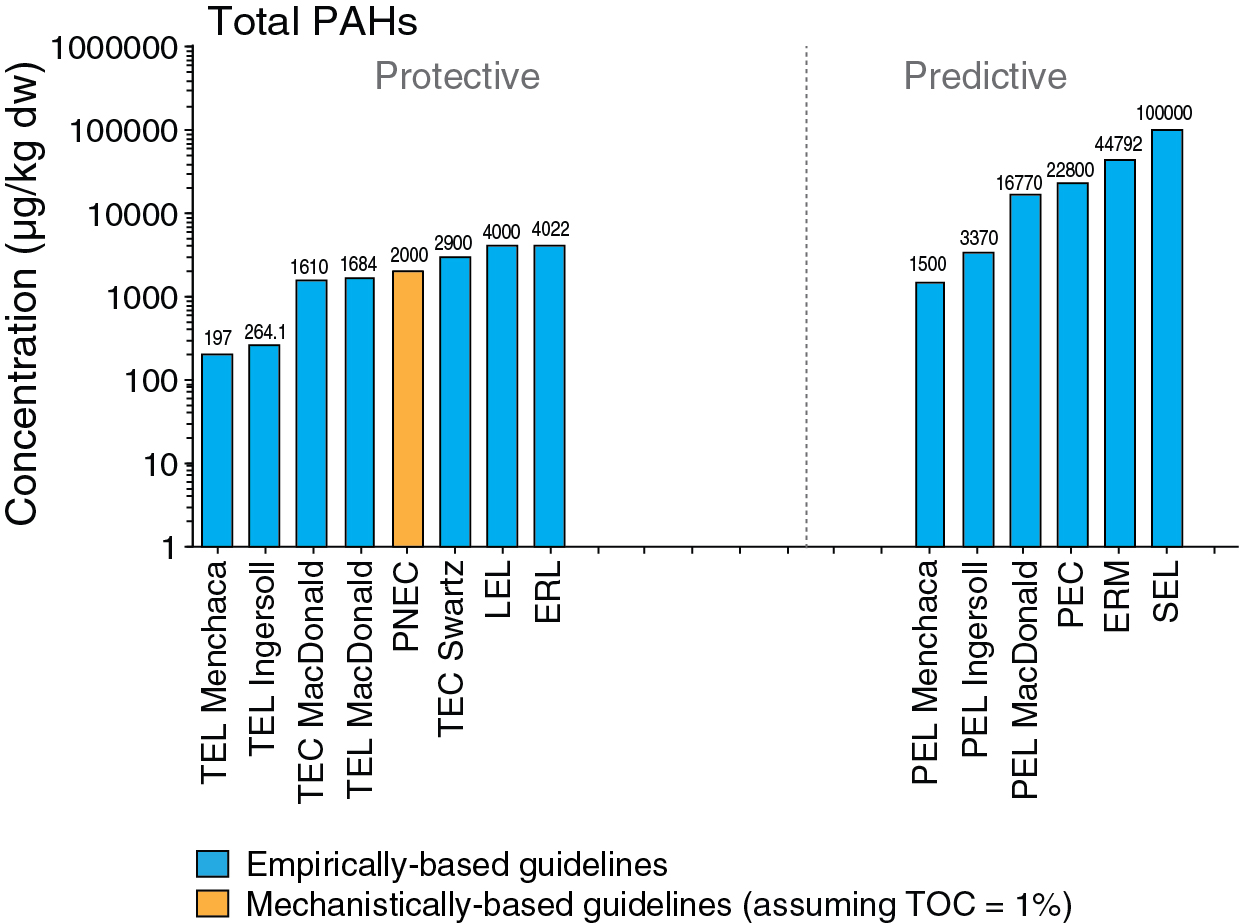


Figure S17. Comparison of guidelines for Total PAHs.
